# High resolution 16S rRNA gene Next Generation Sequencing study of brain areas associated with Alzheimer’s and Parkinson’s disease

**DOI:** 10.3389/fnagi.2022.1026260

**Published:** 2022-12-09

**Authors:** David C. Emery, Maria Davies, Tanya L. Cerajewska, Jelena Taylor, Mae Hazell, Alex Paterson, Shelley J. Allen-Birt, Nicola X. West

**Affiliations:** ^1^Bristol Dental School, Bristol, United Kingdom; ^2^Translational Health Sciences, Learning and Research, Bristol Medical School, Southmead Hospital, Bristol, United Kingdom; ^3^School of Biological Sciences, University of Bristol Genomics Facility, Bristol, United Kingdom

**Keywords:** 16S rRNA NGS, Alzheimer’s, Parkinson’s, brain, oral bacteria

## Abstract

**Introduction:**

Alzheimer’s (AD) and Parkinson’s disease (PD) are neurodegenerative conditions characterized by incremental deposition of β-amyloid (Aβ) and α-synuclein in AD and PD brain, respectively, in relatively conserved patterns. Both are associated with neuroinflammation, with a proposed microbial component for disease initiation and/or progression. Notably, Aβ and α-synuclein have been shown to possess antimicrobial properties. There is evidence for bacterial presence within the brain, including the oral pathobiont *Porphyromonas gingivalis*, with cognitive impairment and brain pathology being linked to periodontal (gum) disease and gut dysbiosis.

**Methods:**

Here, we use high resolution 16S rRNA PCR-based Next Generation Sequencing (16SNGS) to characterize bacterial composition in brain areas associated with the early, intermediate and late-stage of the diseases.

**Results and discussion:**

This study reveals the widespread presence of bacteria in areas of the brain associated with AD and PD pathology, with distinctly different bacterial profiles in blood and brain. Brain area profiles were overall somewhat similar, predominantly oral, with some bacteria subgingival and oronasal in origin, and relatively comparable profiles in AD and PD brain. However, brain areas associated with early disease development, such as the locus coeruleus, were substantially different in bacterial DNA content compared to areas affected later in disease etiology.

## Introduction

This study examines the bacterial presence in the post-mortem brain tissue from two of the most common forms of dementia, Alzheimer’s (AD) and Parkinson’s (PD). There are major differences, but also similarities between these two diseases, including the presence of pathological proteins and significant immune response within specific brain areas, which spread slowly, i.e., over a period of decades, generally from the brain stem upwards to other brain areas. Notably, both AD (Long and Holtzman, 2019; Parnetti et al., 2019) and PD (Noyce et al., 2016; Yilmaz et al., 2019) may have long preclinical and prodromal phases. Of interest in both AD and PD are the factors which initiate and subsequently drive the processes of inflammation, neuronal dysfunction and neuronal death. Many studies in post-mortem human and also animal tissue have suggested microbial involvement (bacteria, viruses and fungi) as potentially causal. Here we attempt to investigate whether, specifically, the bacterial composition present in the brain tissue alters as either disease state progresses, or whether disease is likely to be a direct result of bacterial presence in blood or transfer from the bloodstream. Since the pathological ‘staging’ of these diseases appears to relate directly to their symptomatic progression we have examined, where possible, a range of brain areas which form part of the pathological path from the locus coeruleus in the brain stem to the cerebral cortex.

Alzheimer disease is generally characterized by short-term memory loss, gradually leading to severe cognitive impairment. It is defined post-mortem by its neuropathology which includes extra-neuronal β-amyloid (Aβ) plaques, intra-neuronal phosphorylated tau (ptau), which also comprise neurofibrillary tangles (NFT) deposited in many brain areas including the hippocampus, cortical and subcortical regions. Silver staining of NFT (Braak and Braak, 1991) revealed a consistent spatio-temporal pattern of deposition, starting in the transentorhinal area of the anterior-medial temporal cortex, spreading to the entorhinal cortex (EC), limbic and lateral temporal areas before encompassing much of the neocortex. Further studies however, using different techniques have revealed an increased complexity, with early pathology seen in other areas, in particular the noradrenergic locus coeruleus (LC; Kelly et al., 2021; Ciampa et al., 2022), the cholinergic nucleus basalis of Meynert (nbM) (Schmitz and Nathan Spreng, 2016) and the olfactory system (Esiri and Wilcock, 1984; Ubeda-Banon et al., 2020).

Both amyloid and ptau proteins have been implicated in the process of cognitive decline in AD. NFTs and ptau have a primary association with dementia e.g.(Arnsten et al., 2021); tau pathology is linked to progression of the disease and its passage from the brain stem upwards to the hippocampus and cerebral cortex. Evidence suggests a major initiating role for Aβ in AD, e.g. (Selkoe and Hardy, 2016). Further to this, data provided by positron emission tomography (PET), cerebrospinal fluid (CSF) and plasma Aβ measurements in preclinical AD show increased Aβ levels strongly correlate with decline in episodic memory and executive function (Lim et al., 2020). Additionally, some PET studies suggest that hippocampal ptau may spread to the temporal lobe only in the presence of sufficient Aβ load (i.e., Aβ-positive individuals) (Scholl et al., 2016; Reimand et al., 2020). Notably, amyloid oligomers (AβO), which are capable of activating microglia, have been observed in the LC (Kelly et al., 2021), which is now thought of as one of the likely originating sites of AD pathology. A combination of both Aβ and ptau progression, producing differential but synergistic effects, through amyloid synaptic and ptau axonal degeneration and neuroinflammation, are required for AD dementia (Busche and Hyman, 2020; Pereira et al., 2021). The presence of AβO at the initiation of the disease process may be due to it acting in its known role as an antimicrobial peptide, with resultant microglial induced neuroinflammation.

PD, PD with Dementia, and Dementia with Lewy Bodies (DLB) are all part of a spectrum of synucleinopathy (Dickson, 2012; Donaghy and McKeith, 2014; Kalia and Lang, 2015). This is characterized by intraneuronal deposition of Lewy bodies (LB), comprised of misfolded α-synuclein, a pre-synaptic neuronal protein, which aggregates into pathogenic fibrils. PD is primarily described as a disorder of the motor system with classical symptoms of bradykinesia, rigidity, and resting tremor. LB pathology is seen in the mid-brain substantia nigra-pars compacta (SN) in the early stages of disease, accompanied by dopaminergic cell loss which leads to motor symptoms. Progression of LB pathology into cortical brain regions may result in PD-with-dementia (PDD); conversely, Lewy body dementia (LBD) may occur at first without overt Parkinsonian-like motor symptoms and may later progress towards Parkinsonism. Approximately a third of PDD patients also have cortical Aβ/amyloid-and tau-positive (ptau/NFT) pathology (Smith et al., 2019; Ferreira et al., 2020).

In PD brain, Braak and colleagues described a six-stage disease progression of LB deposition (Braak et al., 2002, 2003a, 2003b, 2004; Del Tredici et al., 2002). Stages 1/2 indicate dual starting points of the dorsal glossopharyngeal-motor nucleus of the vagus (DMV) complex in the lower brainstem and the olfactory bulb. Stages 3/4 describe LB progression to the SN and other midbrain areas, followed by the forebrain. Stages 5/6 are defined by the increasing severity of destruction in cortical areas. A ‘dual hit’ hypothesis was later proposed whereby a pathogen may enter the brain *via* the olfactory bulb or the gastric route (Braak et al., 2003a, 2003b; Hawkes et al., 2007, 2010). More recently it is suggested that this only describes a subset of PD cases, and that LB pathology can originate in areas such as the SN, with the DMV or olfactory bulb unaffected (Kalaitzakis et al., 2008; Rietdijk et al., 2017). Attention has also focused on the noradrenergic LC in the brain stem as an area affected very early in PD as well as AD (Gesi et al., 2000; Grinberg et al., 2011; Del Tredici and Braak, 2016; Weinshenker, 2018; Jacobs et al., 2021; Matchett et al., 2021; Tilley et al., 2021).

Reduced clearance of excess Aβ or α-synuclein, results in inflammation and neuronal death in AD (Kinney et al., 2018; Bruni et al., 2020; Sonninen et al., 2020) and PD, respectively, (Hirsch and Hunot, 2009; Lee et al., 2019; Hirsch and Standaert, 2021). Neuroinflammation, driven primarily by the innate immune system, is mediated through various mechanisms whereby, in AD, genetic risk factors, ageing, and/or prolonged exposure to Aβ have been shown to result in a microglial dysregulated senescent state with impaired phagocytosis and further decreased Aβ clearance (Heneka et al., 2015; Hansen et al., 2018; Boche and Gordon, 2021; Guerrero et al., 2021). This may be followed by an increased inflammatory response driven by cytokine release, including IL1β, through the NLRP3/inflammasome pathway (Kelley et al., 2019), but also with significant contributions from the T-cells of the adaptive immune system (Heneka, 2020). In PD, as in AD, microglial activation and inflammation contributes to pathology (McGeer et al., 1988; Carvey et al., 1991; Mogi et al., 1994; Muller et al., 1998; Nagatsu et al., 2000; McGeer and McGeer, 2004; More et al., 2013; Caggiu et al., 2016; Lecours et al., 2018; Caggiu et al., 2019; Badanjak et al., 2021; Cardinale et al., 2021; de Araujo et al., 2021). Systemic inflammation may result in circumvention of normal blood–brain barrier (BBB) function, which can promote microglial activation *via* peripherally-derived cytokines allowing infiltration of humoral adaptive immune cells into the brain in AD (Kamer et al., 2008; Paouri and Georgopoulos, 2019; Walker et al., 2019) and PD (Fiszer et al., 1994; Brochard et al., 2009; Lee et al., 2009; Long-Smith et al., 2009; Perry, 2010; Ferrari and Tarelli, 2011; Sulzer et al., 2017; Adams et al., 2019; Sun et al., 2019).

Studies over the last three decades have examined links between microbial action and inflammation associated with neurodegenerative processes. As mentioned above, in AD, post-mortem examination, together with information from animal and cell models, have suggested a role for bacteria (Miklossy, 1993; Riviere et al., 2002; Miklossy, 2011; McManus et al., 2014; Miklossy, 2015; Zhan et al., 2016; Emery et al., 2017; Pisa et al., 2020), including several spirochaetal pathogens such as *Borrelia burgdorferi* and periodontal *Treponema* (Riviere et al., 2002; Miklossy, 2015). Other oral or oronasal/bronchial bacteria have also been identified, in particular the periodontal pathobiont *Porphyromonas gingivalis* (*P.gingivalis*) (Poole et al., 2013; Dominy et al., 2019). Furthermore, AD has been associated with *Chlamydophila pneumoniae* infection and the presence of spirochetes (Mawanda and Wallace, 2013; Maheshwari and Eslick, 2015; Fulop et al., 2018). In addition, *Escherichia coli* (*E. coli*) has been detected in post-mortem brain, and was found to be the dominant species in normal control samples (Branton et al., 2013), and increased in AD brain, where it was identified as the pathogenic strain K99 (Zhan et al., 2016). Viruses, such as *Herpes simplex* type 1 (Jamieson et al., 1991; Itzhaki and Wozniak, 2006; Wozniak et al., 2009; Itzhaki, 2021) and Epstein Barr virus (Carbone et al., 2014), as well as fungi (Alonso et al., 2015, 2017; Pisa et al., 2017), have also been linked to AD. Animal models of AD suggest similar associations (Little et al., 2014; Poole et al., 2015; Balin et al., 2018; Diaz-Zuniga et al., 2020).

Studies linking direct CNS infection to PD suggest potential relevance of viral candidates such as *Influenza A* virus and *Herpes simplex* virus type-1(De Chiara et al., 2012) and bacteria such as *C. pneumoniae*. It has been suggested that bacterial factors such as ochratoxin (Sava et al., 2006) or reactivation of actinobacteria or fungal spores (Broxmeyer, 2002, 2017; Berstad and Berstad, 2017), as well as amyloid-like bacterial (or viral) surface proteins (Chapman et al., 2002) may act directly by prion-like propagation of protein pathology (Friedland, 2015; Friedland and Chapman, 2017). Notably, Pisa et al., 2020 described significant differences between fungal and bacterial population profiles in PD post-mortem brains (Pisa et al., 2020). The link between gut dysbiosis and PD reveals differences in gut bacterial microbiome in PD compared with control (Wallen et al., 2020), with LB pathology in the Auerbach’s and Meissner’s plexuses of the enteric system, suggesting a neuronal link between the gut and the brain (Qualman et al., 1984; Wakabayashi et al., 1988; Braak et al., 2006; Long-Smith et al., 2009). Gut inflammation and subsequent gut permeability has been frequently connected with subsequent motor function symptoms in PD (Devos et al., 2013; Hasegawa et al., 2015; Scheperjans et al., 2015; Butto and Haller, 2016). Following this, LB pathology may spread *via* the vagal system to the DMV within the brain (Braak et al., 2003a; Jiang et al., 2017; Lionnet et al., 2018). Bacterial metabolites leading to neuroinflammation (Tremlett et al., 2017; Heiss and Olofsson, 2019) and BBB deterioration (Houser and Tansey, 2017) may also be important factors.

It is now known that Aβ is an antimicrobial peptide component of the innate immune system (Soscia et al., 2010; White et al., 2014; Bourgade et al., 2015, 2016; Kumar et al., 2016; Park et al., 2016; Moir et al., 2018; Barbut et al., 2019; Sampson et al., 2020), providing a mechanism whereby brain microbial presence could contribute to the amyloid cascade process (Selkoe and Hardy, 2016). There is also evidence for an antimicrobial function for *α*-synuclein (Park et al., 2016; Barbut et al., 2019; Sampson et al., 2020).

This study follows and expands upon our earlier 16S rRNA PCR-based NGS study (Emery et al., 2017) of bacterial DNA in human temporal lobe tissue from control and AD brain. Here, we examine specific brain areas associated with different stages of disease development in AD and PD, compared with control brain. We examine relationships between patterns of progressive disease pathology and the presence of specific bacterial populations, and their likely source of origin.

## Materials and methods

### Brain tissue samples

Brain tissue samples (grey matter) were obtained from the South West Dementia Brain Bank (SWDBB), Southmead Hospital, Bristol, BS10 5NB, United Kingdom which has NHS Research Ethics Committee approval to operate as a research tissue bank. Cohort details are given in Supplementary Table S1. Where possible, samples with post-mortem intervals (PMI) of less than 40 h were selected. Sample selection and designation were also based on clinical diagnosis and post-mortem pathology as well as cause of death (C.O.D.).

Next Generation Sequence (NGS) analysis, as performed here, was preceded by polymerase chain reaction (PCR). This was undertaken on frozen human brain tissue samples from the following brain areas: anterior temporal cortex BA38 (AT), entorhinal cortex BA34 (EC), hippocampus (H), locus coeruleus (LC), orbito-frontal/ (lower) pre-frontal cortex BA11 (LF), substantia nigra pars compacta (SN). Some samples of post-mortem tissue were also assessed to examine the effects of PMI, using quantitative, real-time PCR (quantitative polymerase chain reaction, qPCR). Brain areas used for qPCR assessment were: AT, EC, H, LC, LF, SN, additionally dorsolateral cortex (BA9), brain stem (BS-area below LC), middle temporal cortical gyrus (BA21/22) and nucleus basalis of Meynert (nbM).

Approximately 100 mg samples of tissue were thawed under sterile conditions, at which point any visible blood vessels were removed, and the tissue was homogenized. The tube was re-frozen on dry ice and the tissue re-homogenized and suspended in 0.45 ml of T.E. buffer (10 mM Tris pH 8.0, 1 mM EDTA, Sigma Aldrich, St. Louis, Missouri, United States). Total DNA was extracted with 0.5 ml of phenol:chloroform:isoamyl alcohol (PCI; 25:24:1) equilibrated in T.E. buffer and precipitated with 2.5 volumes of ethanol in the presence of 0.2 M NaCl at –20°C overnight. After sedimentation at 17,000 *g* for 10 min the DNA pellet was washed with 70% ethanol and air-dried before being dissolved in 50 μl of T.E. buffer. The entire procedure was carried out under sterile conditions in a laminar-flow hood.

### DNA quantification

Initial DNA concentrations were obtained by A260/280 absorption using a Nanodrop spectrophotometer (ThermoFisher Scientific, Waltham, MA, United States). Samples had a A260/280 ratio of between 2 and 1.8. Double-stranded DNA concentrations were determined fluorometrically using a QuantiFluor^®^ dsDNA System (Promega, Madison, Wisconsin, United States) and a FLUOstar Optima microplate reader (BMG Lab Tech, Offenburg, Germany).

### 16S amplicon libraries

Amplicon libraries were generated by 16SrRNA PCR followed by NGS. The universal rRNA gene V3-V4 primers (Nadkarni et al., 2002) were adapted for use on the Illumina platform (Illumina Inc. San Diego, CA92122 United States), by the addition of the forward and reverse Illumina adaptor sequences: V3 Forward 5′-ACACTCTTTCCCTACACGACGCTCTTCCGATCTTCCTACGGGAGGCAGCAGT-3′ (Tm, 59 ± 4°C) and V4 Reverse 5′-GACTGGAGTTCAGACGTGTGCTCTTCCGATCTGGACTACCAGGGTATCTAATCCTGTT-3′ (Tm, 58 ± 1°C). These primers were designed by (Nadkarni et al., 2002) based on regions of identity within 16S rRNA following alignment of sequences from most of the groups of bacteria in Bergey’s Manual of Determinative Bacteriology (Holt et al., 1994). These were used to generate amplicons under the following conditions: 150**–**200 ng of purified template DNA was combined with forward and reverse primers at a final concentration of 1 μM each, dNTPS (ThermoFisher Scientific) at 200 nM each (final concentration), and 2.5 U of GoTaq DNA polymerase (Promega) with 1× Green GoTaq reaction buffer in a volume of 50 μl. PCR cycle parameters for NGS amplicon generation were: 5 min 95°C, followed by 38 cycles of 30 s at 95°C, 30 s at 65°C, 40 s at 72°C and then 7 min at 72°C. Amplicon purification, quantification of DNA, library preparation and sequencing were carried out by Eurofins Genomics Europe Sequencing GmbH, Jakob-Stadler-Platz, 78467 Constance, Germany using the Illumina 2 × 300 bp paired end platform (Illumina Inc. San Diego, CA 92122, United States).

### Real-time PCR

Real-time PCR conditions and cycle parameters for the amplification of 16S rRNA gene sequences were based upon those previously described (Nadkarni et al., 2002) with the following changes: In a 20 μl reaction, forward and reverse primers were added to a final concentration of 500 nM, along with 100 nM fluorogenic probe and 1× TaqPath qPCR Mastermix (ThermoFisher Scientific). Cycle parameters were: 5 min at 95°C followed by 40 cycles of 30 s at 95°C, then 40 s at 60°C. PCR was performed in a StepOneplus Real-Time PCR system using StepOne software v2.3 (ThermoFisher Scientific). For total bacterial measurements, standard curves used a gel-purified and quantified (QuantiFluor) PCR product were generated using the same primers and *E. coli* DHα1B (K12-derived) genomic DNA template. Standard curve data was always generated on the same PCR plate as sample data and from the same Mastermix. Primers were the universal 16S rRNA gene variable region 3–4 primers described by Nadkarni et al. (2002): 5′-TCCTACGGGAGGCAGCAGT-3′ (forward, Tm, 59 ± 4°C) and 5′-GGACTACCAGGGTATCTAATCCTGTT-3′ (reverse, Tm, 58 ± 1°C) used in combination with the probe 5′-(6-FAM) -CGTATTACCGCGGCTGCTGGCAC-(TAMRA)-3′ (Tm, 69 ± 9°C).

Total human genomic DNA concentrations were first determined using the Quantifluor system, approximately 150 ng of total template DNA was used in each reaction. The exact amount of template DNA was determined by β-globin PCR: Forward 5′-GTGCACCTGACTCCTGAGGAGA-3′; reverse 5′-CCTTGATACCAACCTGCCCAG-3′, combined with the probe 5′-(HEX)-AAGGTGAACGTGGATGAAGTTGGTGG-(BHQ1)-3′, using the reaction conditions as above. Standard curves were generated using purified human genomic DNA extracted from prostate cancer PC3 cells (Kaighn et al., 1979) grown under tissue culture conditions and quantified by the QuantiFluor system. All oligonucleotide primers and probes were manufactured by Eurofins Genomics (Ebersberg, Germany). No-template controls (NTCs) were carried out in the presence of 150 ng of genomic DNA extracted from PC3 cells (Kaighn et al., 1979) grown under tissue culture conditions. However, due to the inhibitory effect of genomic DNA on bacterial amplification, it was not possible to generate sufficient amplicon for NGS analysis in NTCs as bacterial content was too low. Therefore, to produce an NTC for NGS, amplicons were generated with bacterial DNA-free water (Molysis) in the absence of human genomic DNA.

### Sequence data processing

Data was processed in conjunction with Novogene (Beijing) and Eurofins Scientific SE (Luxembourg). Preliminary sequence processing was carried out by in-house scripts (Eurofins) with Illumina chastity filtering, demultiplexing and primer clipping sequences. Paired-end reads (PE) were then merged using FLASH (2.2.00; Magoc and Salzberg, 2011)^1^ using a minimum overlap of 10 bp. Quality filtering included length filtering to remove reads significantly longer or shorter than the expected 445 bp, and the removal of any reads containing ambiguous bases. Chimaeric reads were identified and removed using UCHIME (Edgar et al., 2011) as implemented in the VSEARCH package (Rognes et al., 2016).

### Microbiome profiling

Operational Taxonomic Units (OTUs) were calculated using clustering based on a minimum entropy decomposition (MED) (Eren et al., 2013, 2015; Eurofins, Genomics Europe Sequencing GmbH). Taxonomic information was assigned to OTUs by DC-MEGABLAST alignments of representative OTU sequences with the database:/dbdir/nt.filtered.fa (Release 2019-10-10). Further processing of OTUs and taxonomic assignments was performed using the QIIME software package (version 1.9.1).^2^ Abundances of bacterial taxonomic units were normalized using lineage-specific copy numbers of the relevant marker genes to improve estimates (Angly et al., 2014).

Sequence analysis was also performed by UPARSE software^3^ (UPARSE v7.0.1001; Edgar, 2013) [Novogene (United Kingdom) Company Limited Hong Kong] using all the effective tags and sequences with ≥ 97% similarity assigned to the same OTUs. For each representative sequence Mothur (Schloss et al., 2009) was used against the SILVA SSU rRNA database^4^ (Wang et al., 2007) for annotation at each taxonomic rank (Threshold: 0.8 ~ 1) (Quast et al., 2013). The multiple sequence alignment tool, MUSCLE (Edgar, 2004; Version 3.8.31)^5^ was used to obtain the phylogenetic relationship of all OTU representative sequences. OTU abundance information was normalized by subsampling using a standard sequence number corresponding to the sample with the least sequences. Subsequent analysis of alpha diversity and beta diversity, phylogenetic tree construction and downstream statistical analysis were all performed with this output normalized data.

The Human Oral Database (HOMD; Escapa et al., 2018)^6^ was used to assign potential orally derived taxa. This was compared to SourceTracker2 analysis (Knights et al., 2011) using the source sample training data (faecal),^7^ (subgingival plaque)^8^ and blood (Emery et al., 2021). In order to do this DADA2 (Callahan et al., 2016) was used to merge paired end reads, de-noise, and remove chimeras. Resulting feature tables were de-novo clustered into operational taxonomic units (OTUs) at a 95% identity level. OTUs from “source” samples were used to train SourceTracker2, using QIIME software package (version 1.9.1),^9^ to calculate the proportion of likely sources for our “sink” test brain samples.

### Statistical analysis

Statistical analysis of Alpha and Beta diversity was undertaken. Alpha diversity indices (Chao, Simpson, Shannon, PD Whole tree) were analyzed by Wilcoxon Test for differences between brain areas and disease. Beta diversity was assessed using a number of methods. Weighted and unweighted Unifrac distance were selected to measure the dissimilarity coefficient between pairwise samples (Novogene) and were calculated by QIIME software (Version 1.7.0). Cluster analysis was preceded by principal component analysis (PCA), which was applied to reduce the dimension of the original variables using the FactoMineR package and ggplot2 package in R software (Version 2.15.3). Principal Coordinate Analysis (PCoA) was performed to obtain and visualize principal coordinates from complex, multidimensional data. A distance matrix of weighted or unweighted Unifrac among samples was transformed to a new set of orthogonal axes, by which the maximum variation factor is demonstrated by first principal coordinate, and the second maximum factor by the second principal coordinate, and so on. PCoA analysis was displayed by weighted gene co-expression network analysis (WGCNA), stat and ggplot2 in R software package (Version 2.15.3). Unweighted Pair-group Method with Arithmetic Means (UPGMA) clustering was performed as a type of hierarchical clustering method to interpret the distance matrix using average linkage and was conducted by QIIME software (Version 1.7.0).

In addition to this, four nonparametric statistical methods were used to evaluate bacterial community structural differences between groups which included Analysis of Similarity (ANOSIM); Multi-response Permutation Procedure (MRPP); the nonparametric multivariate test, ADONIS (nonparametric MANOVA), which employs a distance matrix approach and AMOVA.^10^ ANOSIM, MRPP and ADONIS were performed by R software (Vegan package: ANOSIM function, MRPP function and ADONIS function; Community Ecology Package)^11^ (Oksanen, 2022). AMOVA was calculated by Mothur using AMOVA function.

To look for significant differences in the abundance of specific taxa we used *T*-tests to determine differences in bacterial levels between groups and Metastat to assess intra group variation of taxa, where both include *p*-values (significant ≤ 0.05) and *q*-values (corrected for false discovery rate: FDR). Linear discriminant analysis (LDA) with effect size (LEfSe) was used to identify bacteria most likely to be responsible for differences between groups. *T*-tests were conducted by R software; LEfSe analysis was conducted by LEfSe software and Metastat was calculated by R software. *p*-Value was calculated by the Fisher-Pitman Permutation Test (Neuhauser and Manly, 2004) and *q*-value was calculated by method of Benjamini and Hochberg False Discovery Rate (Hochberg and Benjamini, 1990).

### Hemoglobin assay

Hemoglobin measurements were carried out on frozen brain samples homogenized under sterile conditions with Protein Extraction Buffer (1% SDS, 100 mM NaCl, 10 mM Tris–HCl pH 7.6 with phenylmethylsulfonyl fluoride (PMSF) and protease inhibitor cocktail) and centrifuged at 13000 *g* for 10 min. Soluble protein concentration was quantified using the Pierce bicinchoninic acid (BCA) protein assay kit (ThermoFisher.com). Hemoglobin levels were measured using a Hemoglobin Colorimetric Assay Kit (Cayman, Ann Arbor, MI. USA).

## Results

### Sequence data analysis

On average 120,072 sequence reads were generated per sample, 73,474 of which were assigned to OTUs. Sequence counts per sample varied from 26,648 (H147C) to 543,966 (EC949C) with a mean value of 73,474 (Supplementary Table S2; Eurofins). On average, 97% OTU clustering analysis indicated there were 180 OTUs per sample, 97% OTU clustering statistics are supplied in Supplementary Table S3 (Novogene). Species accumulation analysis displayed as a boxplot, processing, read merging, Operational Taxonomic Units (OTU), sequence and MED OTU statistics are summarized in Supplementary Figures S1A,B.

### Relative abundance of taxa

#### Brain areas with oral taxa

**Figure 1:**
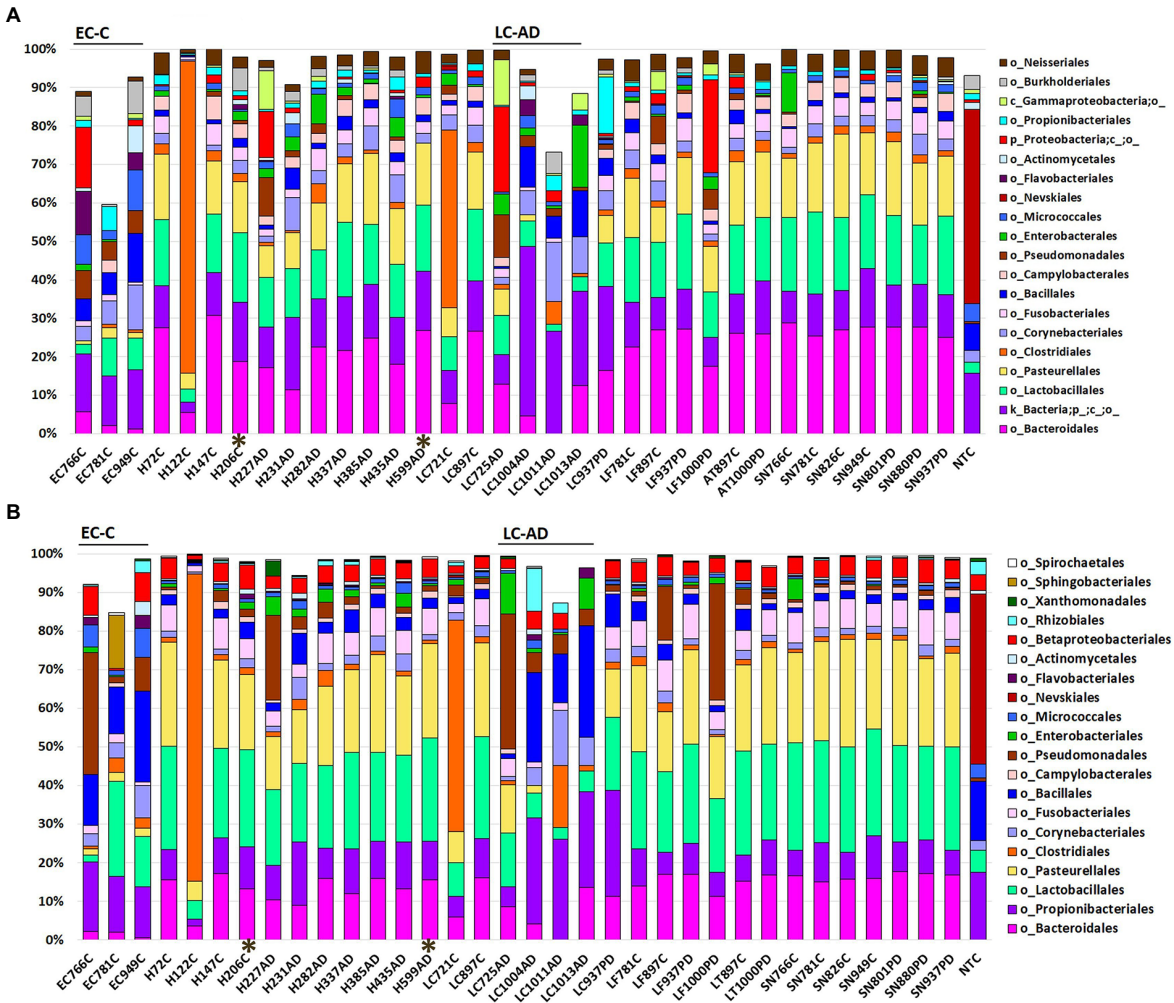
Bacterial population characteristics shown as relative abundance with profiles down to the order level for **(A)** OTUs taxonomically assigned by MED (Eurofins) and **(B)** OTUs taxonomically assigned by 97% OTU clustering as part of the statistical comparison pipeline (Novogene). AD, Alzheimer’s disease; C, control; PD, Parkinson’s disease. AT, anterior temporal pole (BA38); EC, entorhinal cortex (BA34); H, hippocampus; LC, locus coeruleus; LF, lower pre-frontal cortex (BA11); NTC-No Template Control. SN, substantia nigra. ^*^Indicates samples with a post-mortem interval of > 40 h.

Relative abundance profiles for OTUs taxonomically assigned by MED (Eren et al., 2013, 2015; Eurofins; Figure 1A) and by 97% OTU clustering, as part of the statistical comparison pipeline (Novogene; Figure 1B), are given down to order level and, respectively, in Supplementary Tables 4A,B at all taxonomic levels. Top 20 taxa showed that a wide spectrum of the brain areas examined could be characterized at the order level by a consistent combination including Bacteroidales (predominantly oral *Porphyromonas*), Lactobacillales and Pasteurellales. The exceptions to this were EC-C and LC-AD where these three taxa are much less prominent: in fact, *Porphyromonas* was almost entirely absent from both EC-C and LC-AD. Two samples, LC721C and H122C, contained dominant levels of bacterial taxa (e.g., Clostridiales) that did not conform to the patterns displayed by the other members of their respective groups, and samples. PMI intervals for these samples were relatively short (5.5 h and 30 h for LC721C and H122C, respectively). The differences in relative abundancies between the two methods of taxonomic assignment is due to the large ‘uncharacterized’ group in MED, which is discussed further below.

**Figure 2:**
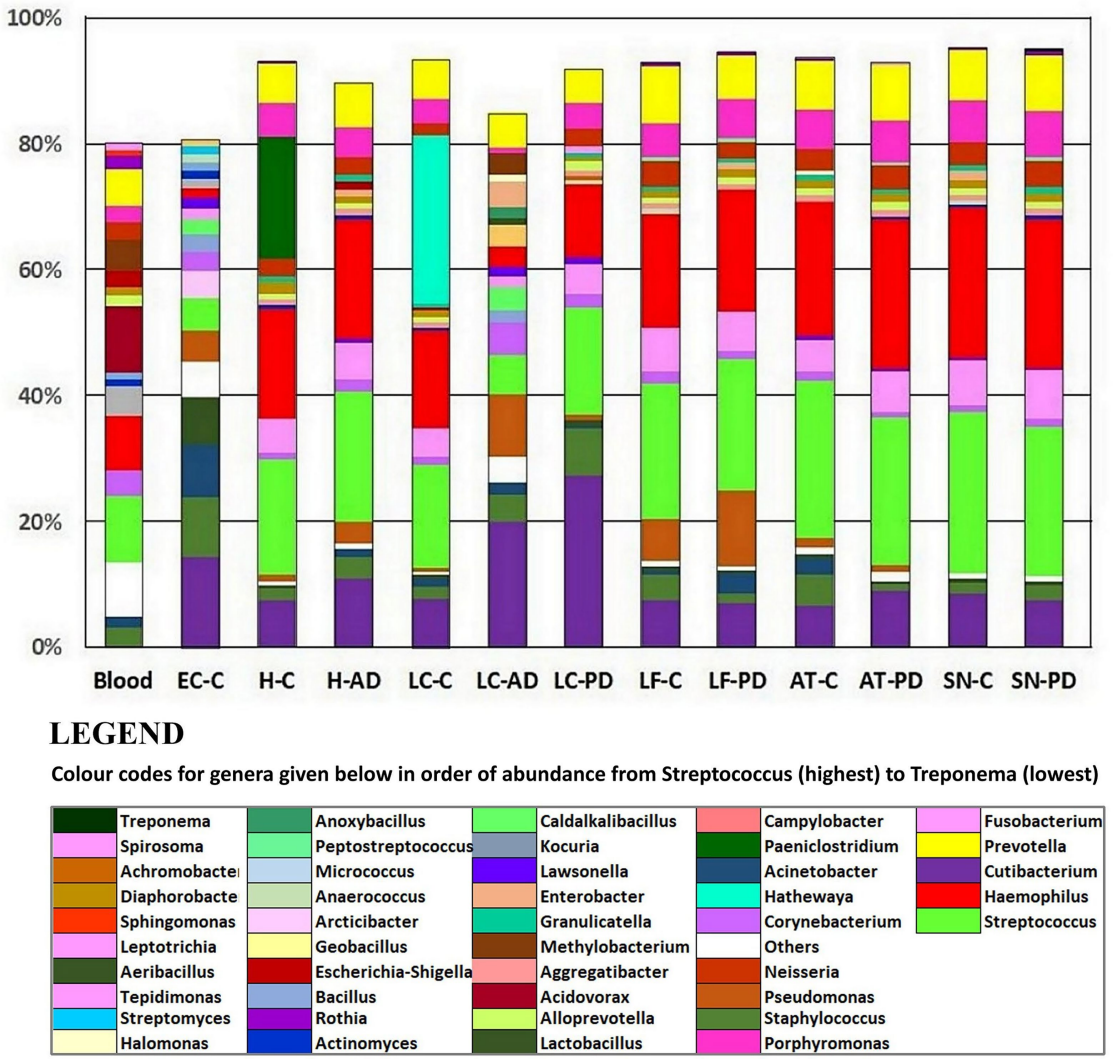
Relative abundances at the genus level using taxonomic assignment based on 97% OTU clustering (Novogene). Included are averaged relative abundance composition of 4 blood samples from a previous study (Emery et al., 2021) which are provided for comparison. AD, Alzheimer’s disease; C, control; PD, Parkinson’s disease. AT, anterior temporal pole (BA38); EC, entorhinal cortex (BA34); H, hippocampus; LC, locus coeruleus; LF, lower frontal cortex (BA11); SN, substantia nigra.

**Figure 3:**
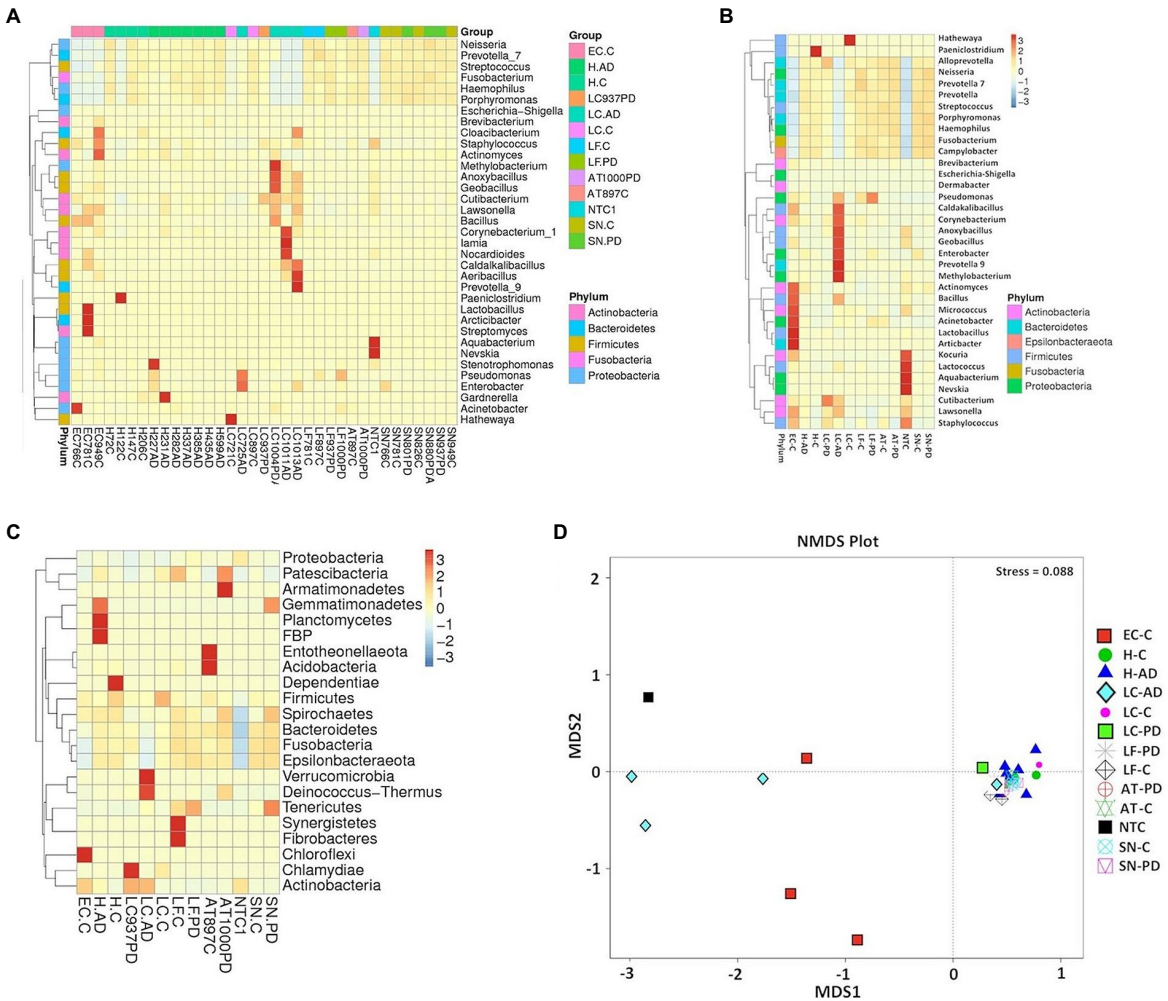
Analysis of bacterial population characteristics based on relative abundance of OTUs taxonomically classified using 97% OTU clustering. **(A)** heatmap cluster analysis (genus) individual sample, **(B)** heatmap cluster analysis (genus) group, **(C)** heatmap cluster analysis (phylum) group, **(D)** Group analysis by Non-Metric multi-Dimensional Scaling (NMDS). Abbreviations: AD – Alzheimer’s disease, C – control, PD – Parkinson’s disease; AT - anterior temporal cortex (BA38); EC - entorhinal cortex (BA34); H - hippocampus; LC - locus coeruleus; LF - lower pre-frontal cortex (BA11); NTC - No template Control; SN – substantia nigra.

Taxonomic assignment at genus level is shown in Figure 2 and Supplementary Tables S4B and S5; while cluster heatmap analysis based on relative abundance is shown in Figures 3A–C. This data was consistent with order level abundance data (Figures 1A,B), showing that, at the genus level, the hippocampus (H-C and H-AD), lower prefrontal cortex BA11 (LF-C), locus coeruleus from control (LC-C) and PD (LC-PD) donors (but not LC-AD), anterior temporal lobe (AT-C, AT-PD) and the substantia nigra pars compacta (SN-C, SN-PD) could be characterized by a cluster of nine bacterial genera: *Alloprevotella*, *Neisseria*, *Prevotella 7*, *Prevotella*, *Streptococcus*, *Porphyromonas*, *Haemophilus*, *Fusobacterium* and *Campylobacter* (Figure 3B), all of which can be classified as oral using the Human Oral Microbiome database (HOMD) (Escapa et al., 2018). This analysis also showed that EC-C and LC-AD contained much lower levels of these oral taxa and, instead, were characterized by their own clusters, neither of which were primarily oral. Non-linear multidimensional scaling (NMDS) cluster analysis is consistent with this pattern with all high oral content brain groups clustering together, while EC-C and three of the LC-AD samples grouped separately (Figure 3D). The exception to this was LC725AD which had moderate levels of the oral group of 9 taxa and clustered with the high oral samples. In fact, sample LC725AD was different to the other members of its group in several respects (see below).

*Porphyromonas*: 97% OTU clustering taxonomic assignment suggested *Porphyromonas* content to consist almost exclusively of two species, *P. gingivalis* (strain TDC60) and *P. endodontalis*, with *P. gingivalis* (TD60) always greater in abundance at a ratio of ~1.6 (Supplementary Table S4B). MED identified *P. endodontalis*, as the main strain (Supplementary Table S4A).

Spirochaetes: Heat map analysis at the phylum level indicated that Spirochaetes were also a consistent, but relatively minor component of the oral cluster, with highest level in AT-PD and SN-PD samples (Figure 3C).

#### ‘Uncharacterized’ OTUs

**Figure 4:**
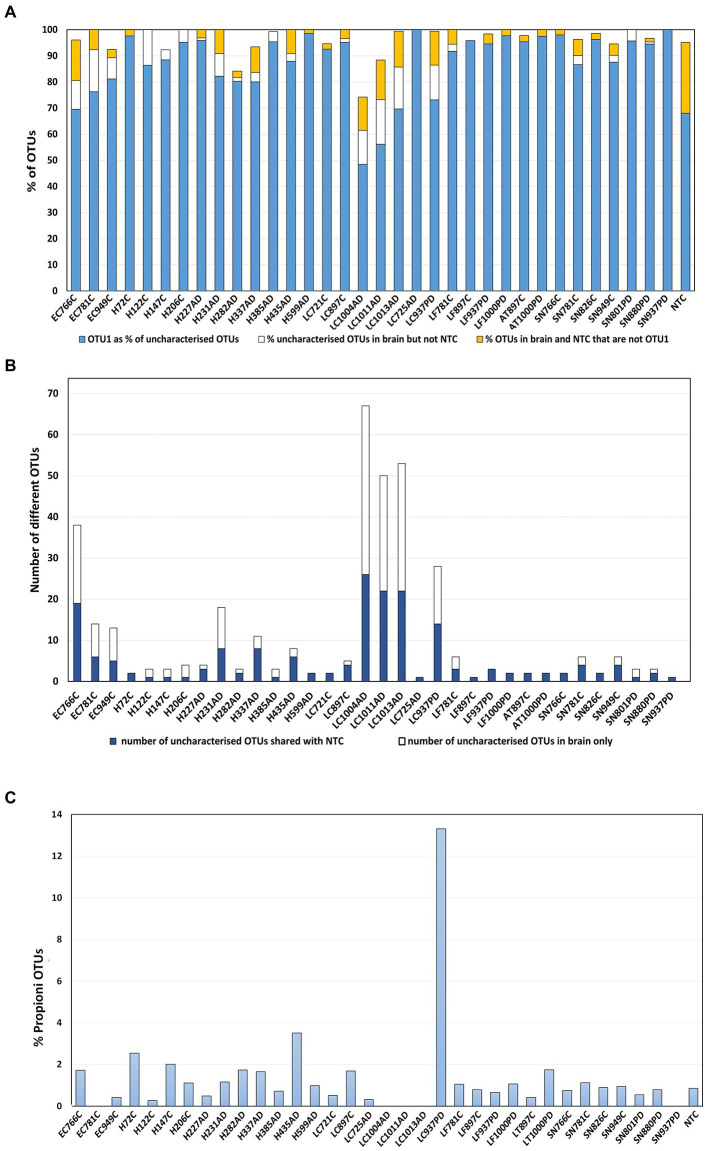
Uncharacterized OTUs. **(A)** OTU1 as a percentage of the total uncharacterised OTUs per sample shown in blue, this was defined by MED as ‘Kingdom’ (k_bacteria;p), attributed by BlastN analysis as *Cutibacterium acnes*. In gold: the percentage of uncharacterised OTUs per sample that are not OTU1 and are in both brain and NTC. In white: the percentage of uncharacterised OTUs per sample that are not OTU1 that are in brain only. **(B)** Numbers of different uncharacterised *Cutibacterium* OTUs per sample only in brain (blue); number in brain and also in NTC (white) **(C)** Relative abundance of f_ *Propionibacteriacea* overall in each sample. 12 OTUs were defined to family (f) Propionibacteriaceae; BlastN analysis showed close correspondence of these to *Cutibacterium granulosum*.

The category ‘Kingdom’ (k_bacteria; p) as defined by MED (Eurofins), hence-forth referred to as ‘uncharacterized group’, was a major component in all samples (Figure 1A) and contained various combinations of 164 different uncharacterized OTUs. These were dominated by OTU 1 (87%) which BlastN analysis attributed to *Cutibacterium acnes* species (*C. acnes* formerly *Propionibacterium acnes*). 97% OTU clustering (Novogene) automatically assigned this OTU as genus *Cutibacterium* (Supplementary Table S4B)*,* with the *Cutibacterium* total relative abundances for LC-AD (except LC725AD), LC-PD and EC-C noticeably higher than the rest (Figure 2; Supplementary Tables S4B, S5). It is also worth noting here, that although the presence of *C. acnes* in NGS data should always be interpreted with caution (Mollerup et al., 2016), the results here are not homogenous, as would be perhaps expected by contamination, but fit into a pattern. Further, the *Cutibacterium* component of brain samples does not substantially overlap with NTC (Eurofins; Figures 4A,B), and clustal Omega sequence alignment of all uncharacterized *Cutibacterium* OTUs (Supplementary Figure S2) indicates substantial clustering of OTUs which are exclusive to AD brain. Notably, a high level of *C. acnes* (aka *P. acnes*) was seen in our previous study of brain tissue (Emery et al., 2017).

In addition to *C. acnes* sequences, a group of 12 OTUs taxonomically defined to family (f) Propionibacteriaceae by MED, corresponded to *Cutibacterium granulosum* (Figure 4C) as defined by BlastN analysis. These appear in most samples at, on average, 1%, including the NTC, but interestingly, were present in sample LC937PD at ~ 13% (Figure 4C).

### Alpha diversity

**Figure 5:**
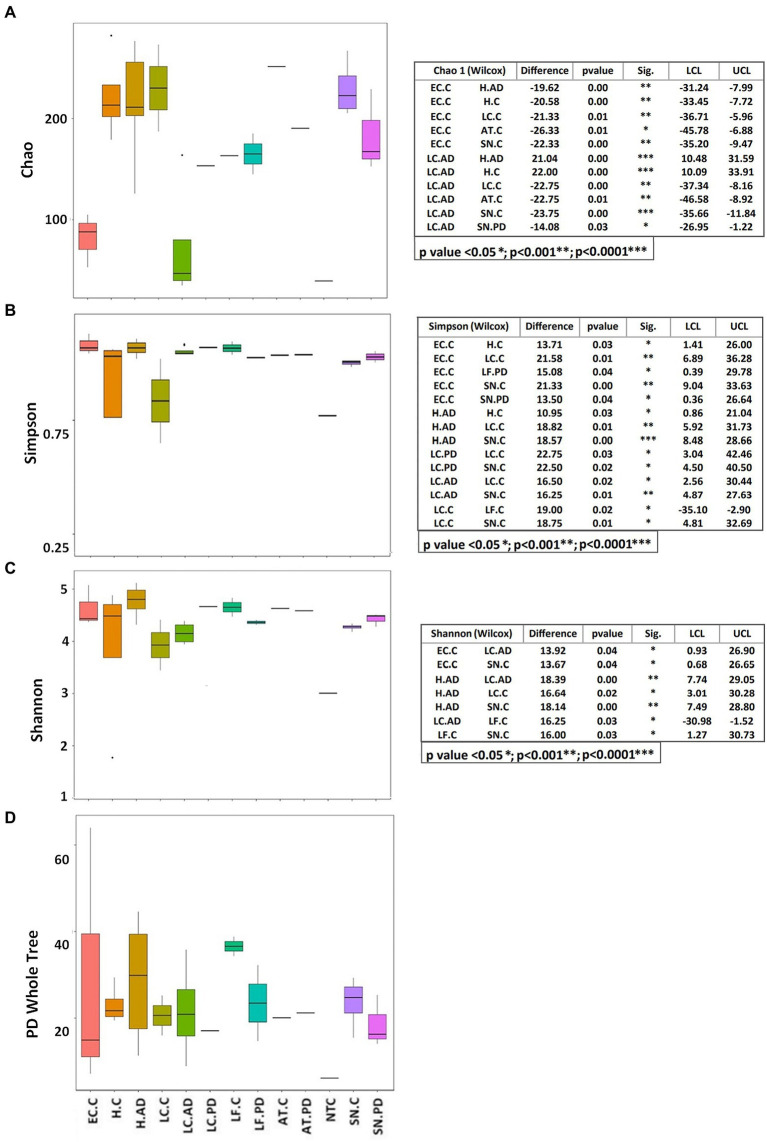
Alpha diversity indices displayed as boxplots with significant differences between groups calculated by the Wilcoxon test. **(A)** Chao, **(B)** Simpson, **(C)** Shannon, **(D)** PD (phylogenetic diversity) whole tree. AD, Alzheimer’s disease; C, control; PD, Parkinson’s disease; AT, anterior temporal cortex—BA38; EC. entorhinal cortex (BA34); H, hippocampus; LC, locus coeruleus; LF, lower pre-frontal cortex (BA11); NTC, No template Control; SN, substantia nigra.

The apparent differences between EC-C/LC-AD and the rest of the brain regions/samples indicated by relative abundance profiles are further emphasized by measures of alpha diversity (Figure 5), especially richness (Chao1; Figure 5A) which is significantly lower in these two groups compared to the rest. Simpson and Shannon diversity functions also show significant differences between groups (Figures 5B,C). Most frequently these are between EC-C and all areas except H-AD, but also between disease groups and controls including H-AD v H-C. Phylogenetic diversity (Figure 5D) showed no significant differences between groups, but there was a notable increase in the ranges in EC-C and H-AD, LC-AD (compared to LC-C) and LF-PD (compared to LF-C).

### Beta diversity

**Figure 6:**
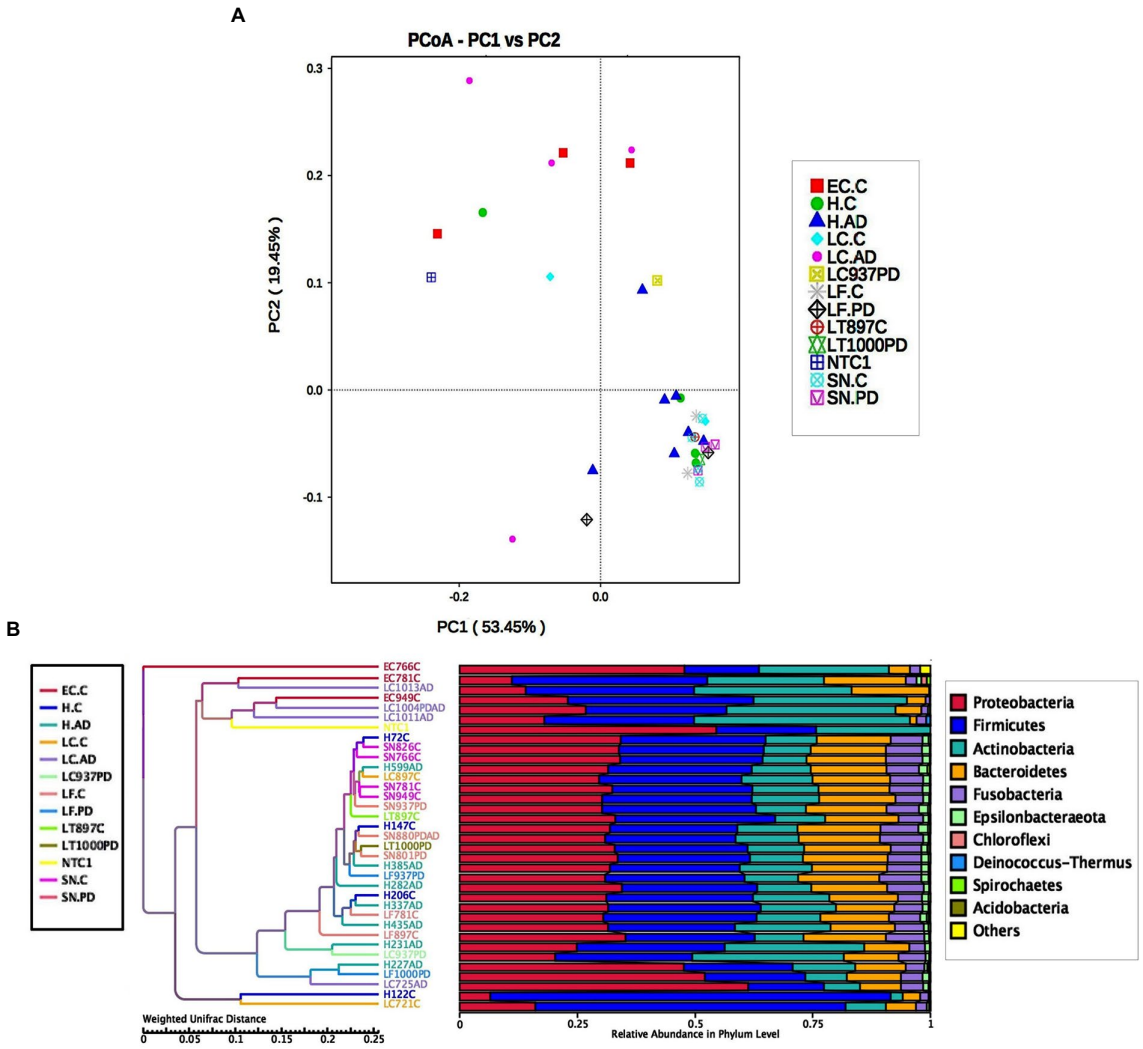
**(A)** PCoA cluster analysis of weighted Unifrac distances. **(B)** Cluster tree analysis using UPGMA Unweighted Pair-group Method with Arithmetic Mean (weighted Unifrac).

Significant differences in unweighted Unifrac distances between groups were confined to those pairings containing either EC-C or LC-AD while weighted Unifrac distances revealed significant differences between most groups (Supplementary Figure S3). Weighted Unifrac distances are also depicted by PCoA cluster analysis (Figure 6A), UPGMA (Figure 6B) and pairwise phylogenetic matrix heatmap (Supplementary Figure S4). Those same samples characterized by nine potentially oral genera (groups H-C, H-AD, SN-C, SN-PD, LF-C, LF-PD, AT-C and AT-PD) in the clustered heatmap analysis are mostly grouped together (and separately from LC-AD and EC-C) in PCoA. Supplementary Figure S4 also shows how samples LC721C and H122C are phylogenetically more distant from their respective groups due to their Clostridiales content (*Hathewaya* and *Paeniclostridium* respectively) even though they contained the same group of oral taxa.

### Community variance statistics

Differences in bacterial community structure between groups were also evaluated by four nonparametric methods, ADONIS, AMOVA, ANOSIM and MRPP. These results are summarized in Supplementary Table S6. These are in good agreement both with each other and the patterns seen in alpha diversity and OTU relative abundance, all showing significant differences between EC-C or LC-AD and most other groups, the most common significant differences being between EC-C and H-C or SN-C and between LC-AD and H-AD.

### Between-group variation analysis of specific taxa

**Figure 7:**
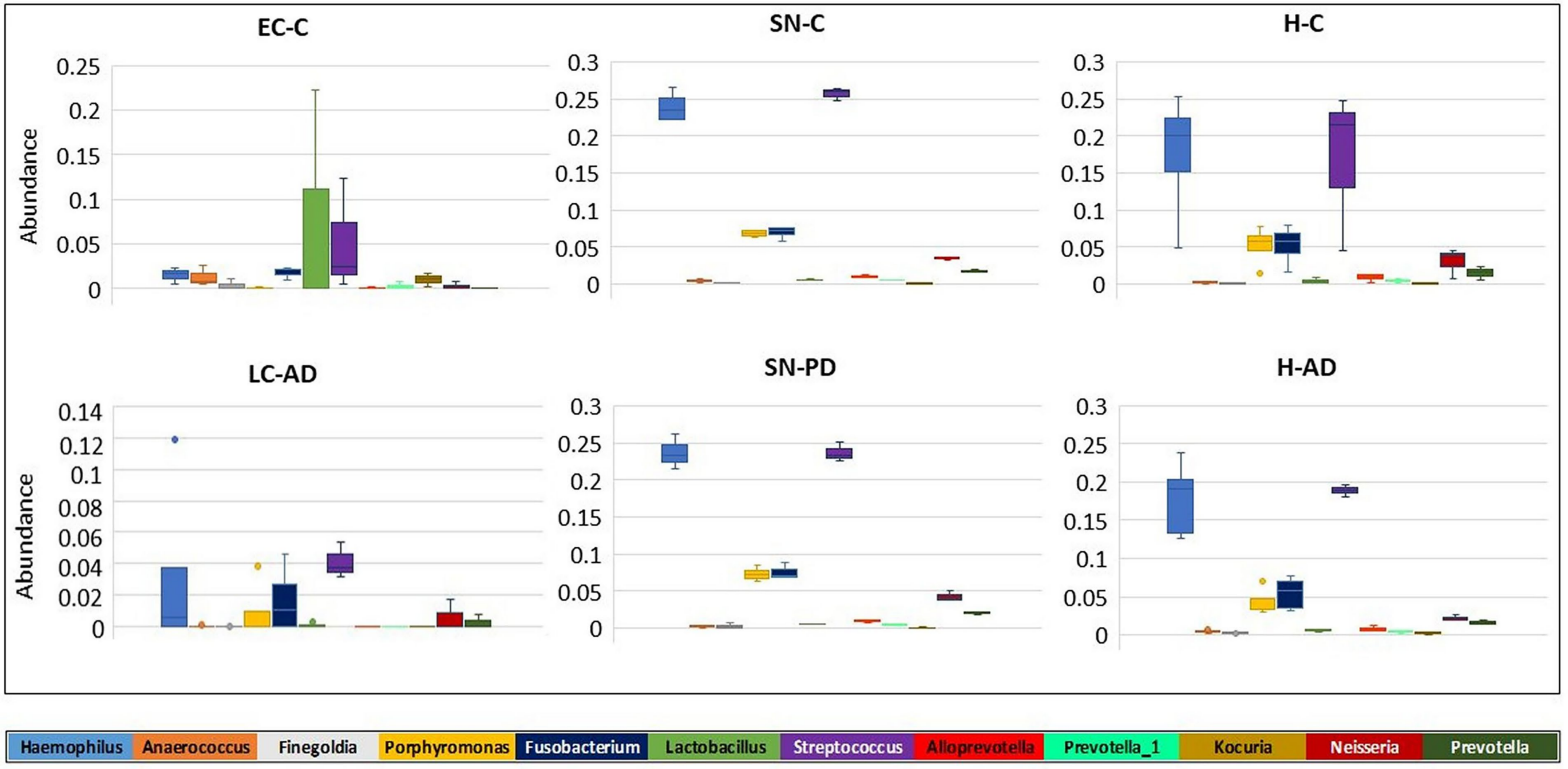
Metastat analysis of the differences in intra-group variation in taxa between groups. The genera (top 12) with the greatest differences in intra-group variation between groups are shown. Significant differences in genera variation were seen between H-AD and LC-AD groups for all genera shown (Supplementary Table S7 gives *p* and *q* values).

**Figure 8:**
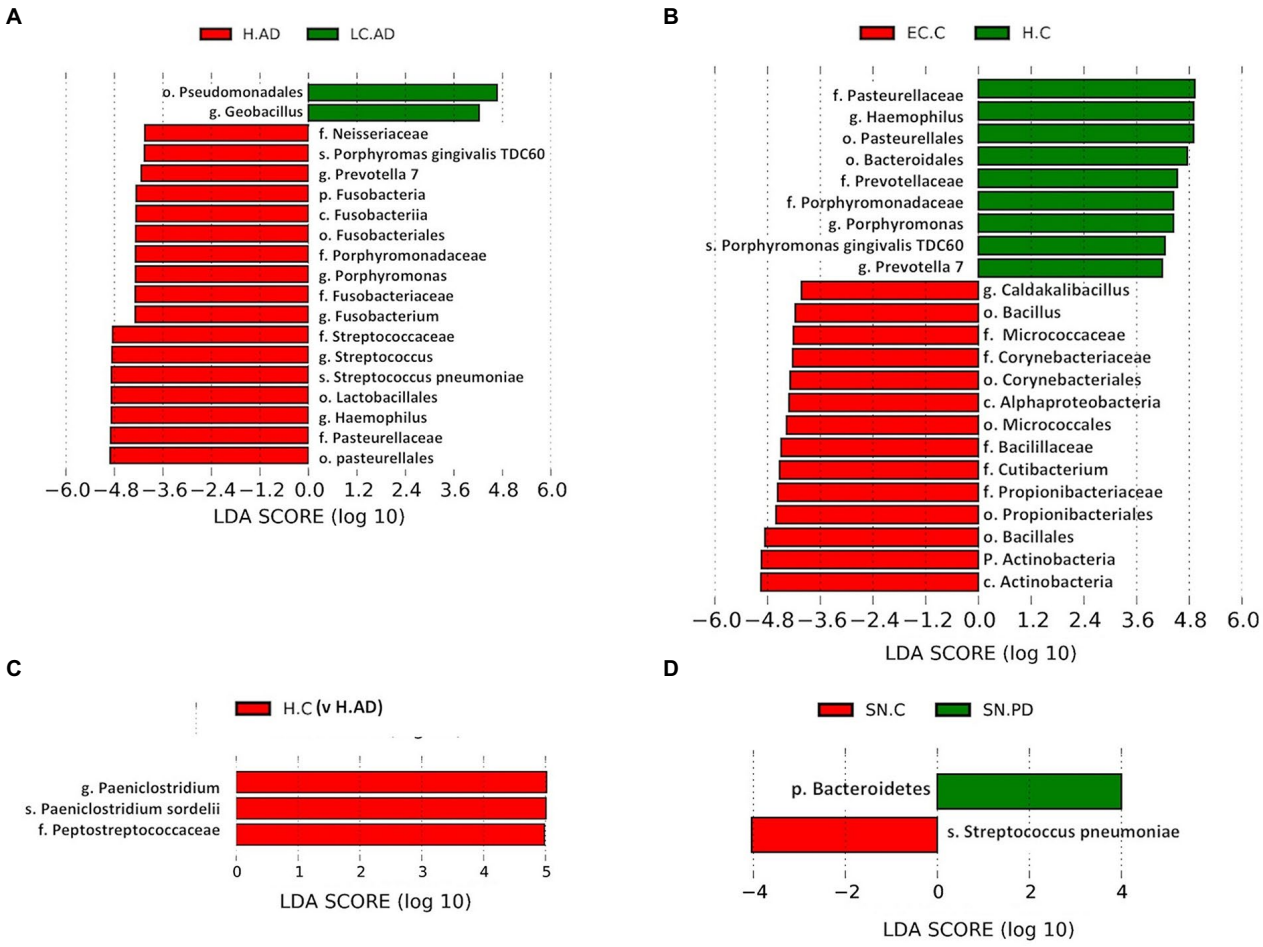
**(A-D)**: LEfSe analysis of differentially abundant taxa. *T*-tests highlight taxa abundance significant differences with four pairings: H-AD/LC-AD **(A)**; H-C/EC-C **(B)**; H-AD/H-C **(C)**; and SN-C/SN-PD **(D)**. C, Control; AD, Alzheimer’s disease; PD, Parkinson’s disease; EC, entorhinal cortex; H, hippocampus; LC, locus coeruleus; SN, substantia nigra.

Significant differences in taxa between groups as determined by T test, Metastat analysis and LEfSe analysis are summarized in Supplementary Table S7 and given in full in Supplementary Tables S8,S9. Notably, T-tests highlight taxa abundance differences mostly between LC-AD and H-AD, with a limited number of significant differences between EC-C and H-C and none between H-C vs. H-AD and SN-C vs. SN-PD. Metastat analysis was consistent with this, with significant differences only detected between LC-AD and H-AD (Figure 7) and the top 12 genera differences common to those highlighted by T-test. LEfSe analysis (Figures 8A–D) found significant differences between four pairings: EC-C/H-C, LC-AD/H-AD, H-C/H-AD and SN-C/SN-PD. H-C was differentiated from H-AD by *Paeniclostridium* that is overrepresented in a single sample (and may not be relevant to disease) and by oral Peptostreptococcacae. SN-C and SN-PD were differentiated from each other by higher levels of (p) Bacteroidetes in SN-PD and *Streptococcus pneumoniae* (*S. pneumoniae*) in SN-C. H-C and H-AD were both characterized by oral taxa whereas EC-C and LC-AD were not, as indicated in the heatmap cluster analysis (Figure 3).

### Blood content of samples

Hemoglobin levels showed that, apart from two samples, SN880PD (42.9 mg hemoglobin/mg of total soluble protein) and AT781C (41.8 mg hemoglobin/mg), average levels were 5.6 mg hemoglobin/mg of total soluble protein (with SN880PD and AT781C removed). Whole blood contains 120–180 mg hemoglobin/ml (Lodemann et al., 2010). By this measure brain extracts were approximately 3.7% blood-derived. Samples SN880PD and AT781C showed no obvious differences in bacterial population profile to the other brain samples. Supplementary Table S10A shows the top 20 genera from each brain group, alongside that of blood, and indicates that 45–65% of genera were common to both blood and brain groups. The relative abundance of these, however, differed considerably between brain and blood (Figure 3). For instance, the low oral content brain groups contained around half the *Streptocococcus* levels seen in blood (5.1% in EC-C and 6.3% in LC-AD compared to 10.6% in blood) and considerably less *Haemophilus* (1.5% in EC-C and 3.2% in LC-AD compared to 8.5% in blood). In contrast, high oral brain groups averaged 22.3% *Streptococcus* and 20.1% *Haemophilus*. Three blood genera, *Acidovorax* (10.4%), *Tepidomonas* (1.2%) and *Sphingomonas* (0.9%) were not present in brain samples.

### Origins of brain-derived bacterial DNA

**Figure 9:**
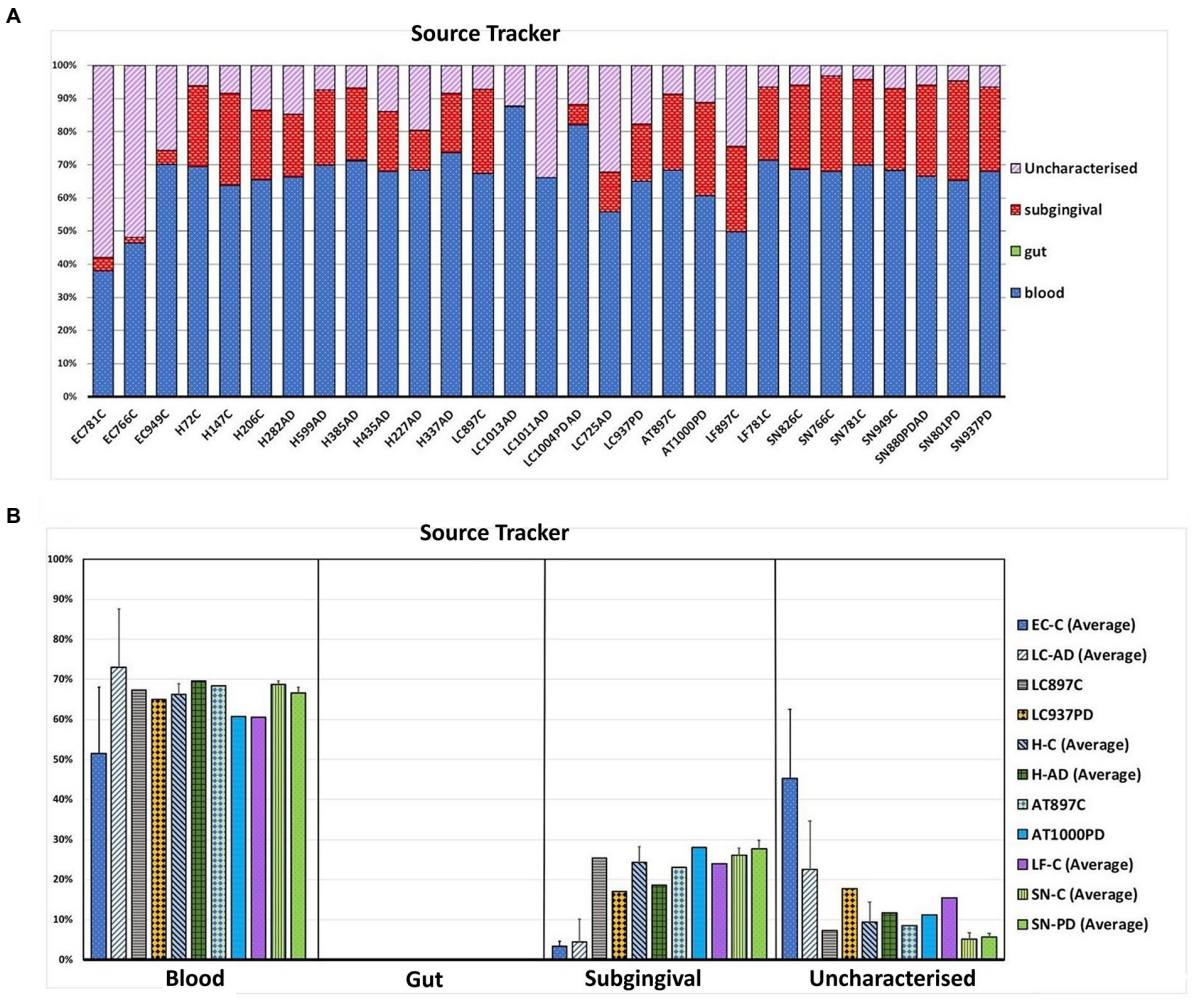
Source tracking using blood, gut and subgingival tissue as training sets for the microbial profiles of these areas. **(A)** Proportions of bacteria matching each source in individual brain samples. **(B)** Averaged values (where applicable) per group per predicted source. Bacterial sequences in the brain samples are shown to be similar at 65.2% on average to the blood training set. No gut content of brain samples was seen, however, a number of bacteria in brain samples did not match the training sets and were thus denoted as uncharacterized.

Shown in Supplementary Table S10B are the averaged top 20 genera for each brain group and that of blood with taxa listed in the HOMD as oral or nasal highlighted in orange. Oral content in the brain varies from 24.4% (LC-AD) and 31.5% (EC-C) to 84.3% in SN-PD. This compares to blood which, by this measure, contained 40.7% oral bacterial DNA. SourceTracker analysis (Figure 9) used three training data sets: blood, gut, and subgingival microbiomes. Comparison of our bacterial profiles with those of the datasets indicated that on average 65% of bacterial taxa detected here in brain are also present in the blood data training set. It also indicated that the subgingival content in EC-C (3.3%) and LC-AD (4.5%) was lower than all other areas (average 23.8%). Taxa of uncharacterized origin which did not match any of the training datasets were higher in EC-C and LC-AD compared to the rest and represent mainly skin and oro/nasal taxa (*Cutibacterium*).

### Effect of PMI assessed by real-time PCR

Supplementary Figure S5 shows that for most cortical and sub-cortical regions sampled neither disease state nor post-mortem interval (PMI) were associated with 16S rRNA DNA levels. The exception to this was the temporal BA21/22 region which contained extreme levels of 16S rRNA DNA in many samples with a PMI of ≥ 40 h and was, therefore, excluded from the NGS study. Where possible, samples used in the NGS study had PMIs of less than 40 h. Only three samples were above this: 42, 59, and 72 h.

## Discussion

### Overview

This 16SrRNA NGS study examines the nature and distribution of bacterial DNA in human post-mortem brain samples in AD and PD, compared to controls. Brain areas included those affected in early, intermediate and later stages of pathology: LC in the pons, SN in the midbrain, hippocampus, entorhinal cortex (EC/BA34), and various cortical regions including AT/BA38, and LF/BA11. Other microbials such as fungi or viruses are not investigated here; however, a comprehensive study of the human microbiome in tissues including the brain has been recently published (Hu et al., 2021).

In the present study, profiles of control, AD and PD were generally similar, containing predominantly oral species, in most donors these were in those brain areas which are usually affected by AD/PD early/mid-phase pathology. Interestingly, a lower oral content was present in LC-AD and EC-C. LC-AD was substantially different to LC-C, with much lower levels of oral taxa; LC-AD, LC-PD, and also EC-C had the highest levels of *Cutibacterium* (14.4–27.4%), the greatest component of which was determined as *C. acnes.* Furthermore, the EC also had substantially different bacterial profiles to all other areas including the adjacent hippocampus. The profile of bacteria in the brain was also distinct from that found in blood.

As mentioned earlier, many studies in human brain tissue and animal models of AD have shown links with various bacteria, fungi and viruses (Miklossy, 1993; Pisa et al., 2017; Itzhaki, 2021). Of these, bacterial species such as spirochaetes, for example *Borrelia burgdorferi* and periodontal Treponema; oral/oronasal/bronchial bacteria such as *P.gingivalis* and *C. pneumoniae* have been detected (Riviere et al., 2002; Poole et al., 2013; Miklossy, 2015; Dominy et al., 2019). In this present study we are not able to propose specific bacteria as a direct cause of disease, i.e., with no presence in control tissue. However, many of the bacterial species detected here are capable of causing disease under certain circumstances. If the presence here, of any of these bacteria are related to disease, the relationship might require a specific bacterial load or a particular mix of different bacteria in determined proportions. For instance, tipping the balance towards disease may require brain areas to support hierarchies, as seen in subgingival tissue in periodontitis, and/or the ability to excite neuroinflammation, which may result in neuronal death in some subjects, but not others. This study provides relative proportions of bacterial species and thus can detect differences in relative amounts of taxa that are present, unless abundance is at very low levels. Due to the PCR amplification of 16SrRNA to provide the material for NGS we are unable to calculate exact numbers of bacteria present. However, with reference to the qPCR (Supplementary Figure S5) which includes samples from the NGS dataset, bacterial levels in the samples from the brain areas were in the region of 5,000 copies 16SrRNA/100 ng template.

Since we have found bacterial profiles within the brain tissue of controls and those with disease, that are somewhat different from blood profiles, it is likely that these bacteria can enter the brain parenchyma by other means, for instance *via* the olfactory tract. It would be important to be able to understand for instance, whether specific antibiotics would be able to reduce symptoms of mild cognitive impairment. It has recently been suggested that mid-life use of antibiotics results in cognitive impairment in later life (Mehta et al., 2022) possibly due to destruction of gut microflora; however, the administration of these antibiotics was presumably due to the presence of bacteria, because of an overt infection, but which may, unbeknownst, already have also begun to penetrate the brain. In accord with this, the results of a recent Swedish study suggest that infectious events may trigger or accelerate a pre-existing disease process, leading to an early clinical onset of neurodegenerative disease (Sun et al., 2022).

### Confounding factors

The characterization of bacterial metagenomic data from human post-mortem brain samples is constrained by three main putative confounding factors: (i) contaminating bacterial DNA and live bacterial cells from within blood vessels, (ii) post-and peri-mortem growth of bacteria and (iii) the extremely low biomass of bacterially-derived DNA in an overwhelming background of host genomic DNA. This is discussed below:

#### Contributions from blood

Although there were similarities between the blood and brain profiles, the blood bacterial content profile was clearly not replicated in the brain tissue. This is demonstrated by the Top 20 genera profiles from blood microbiome data (Emery et al., 2021) compared to brain region profiles as shown in Supplementary Table S10A. Notably, the second largest blood component, *Acidovorax*, consistently present on average at 10.4%, is seen in only one brain sample (LF897C, 0.3%; Supplementary Table S4A). Similarly, *Tepidimonas* was consistently present in blood at on average 1.2% and *Sphingomonas* at on average 0.9%, both of which were absent from all top 20 genera in brain profiles. Conversely, brain samples consistently contained a group of ~10 genera in their ‘top 20’ profiles which were not present in the blood ‘top 20’, including *Fusobacterium*, *Aggrigatibacter*, *Granulicatella*, *Lactobacillus*, *Lawsonella*, and *Peptostreptococcus*. Differences in bacterial profiles in the brain CSF and blood from patients with bacterial meningitis have also been reported recently, for example *Delftia acidovorans* was only identified in blood, whereas only CSF contained *Pseudomonas sihuiensis*, and there were clear differences in the relative abundance of other species (Liao et al., 2021). Furthermore, hemoglobin measurements that indicate the contribution of blood vessels within brain tissue is very small in almost all samples suggesting that significant differences in microbiome profiles between brain areas/disease are not the result of differences in blood content. Together, these factors suggest that the blood content of blood vessels within brain samples is likely only to be a minor contributor to these data. Nevertheless, at genus level there is some overlap between the blood and brain bacterial population profiles shown here which must be considered when interpreting the data.

#### Post-mortem interval

Post-mortem interval also considered here as a possible confounder. Quantitative PCR measurements of bacterial load from an overlapping, but separate study (Supplementary Figure S5), suggest that PMI did not significantly affect bacterial DNA levels in most brain areas examined. An increase in bacterial level appeared only to be a factor in the temporal BA21/22 region out of a total of ten brain areas, and only in those of over 40 h PMI This area is particularly affected in the latter stages of AD and may be more vulnerable to contamination from the gut and blood. Nevertheless, this does not inform as to whether there is an increase in some types of bacterial populations and not others. The number of samples assessed for NGS here does not allow us a definitive answer to this question, however in bacterial population characteristics shown as relative abundance down to the order level, two samples have > 40 h post-mortem (H206C and H599AD), yet neither of these differ in substance from the rest. The two which are different however, (L721C and H122C), both had high levels of Clostridiales present and have reasonably low post-mortem times (5.5 and 30 h). These are bacteria which are generally known to be attributable to pre-mortem infections; perhaps related to lengthy use of antibiotic medications before death; clinical data was unfortunately not available for all subjects.

#### PCR amplification

A further methodological factor to be considered is that of PCR amplification of extremely low biomass target sequences, which is challenging especially in the context of high levels of genomic host DNA (Kebschull and Zador, 2015; Bedarf et al., 2021). This is especially true for non-specific 16SrRNA gene amplification using 16S gene constant region primer sites, due to the relatively high degree of similarity to human 18S sequences. To counter this we have developed a highly efficient methodology of extracting and amplifying bacterial (and non-host DNA fragments in general) requiring only one 38 cycle round of PCR, thus avoiding distortions in measured population abundances associated with excessive PCR cycles (Kelly et al., 2019). We found the primer pair and PCR conditions used here (Nadkarni et al., 2002) to be optimal for several reasons: 16SrRNA sequences are amplified with high efficiency and have been shown to have high taxonomic coverage; they offer good differential priming sequences to human 18S for an amplicon containing two variable regions and, although in the absence of 16SrRNA sequences they do amplify extraneous sequences (mostly 18S-unpublished data), the variable region 3–4 amplicon is approximately 120 bp smaller than the equivalent 18S product and is easily gel-purified during library preparation. Under the conditions described here, however, an 18S PCR product is not seen when bacterial DNA is present. It may be of importance to clearly state here, in that SDS (sodium dodecylsulfate) lysis buffer was not used in extraction. If used, microbial DNA can be overwhelmed by the inordinate quantity of human genomic DNA, making its detection difficult.

#### Non methodological confounders

In addition to the methodological confounders, three additional non-methodological confounders need to be considered. All brain material was sourced from the SWDBB, which relies on regional donations; therefore, data obtained cannot automatically be extrapolated to the whole population. It is also accepted that the donor numbers presented here are small, unfortunately the covid pandemic precluded adding to the dataset, and delayed NGS analysis. Finally, the SWDBB can only provide details of known co-morbidities, other unreported comorbidities may be present affecting the bacterial profiles. Despite these potential confounders, clear patterns in bacterial profiles were observed which were not common across all samples/areas analyzed and provide an insight into bacterial communities in different brain regions.

### Differences in bacterial 16S DNA profiles between groups

#### Abundance

Tissue was examined in control, AD and PD brain including AT/BA38, LF/BA11, hippocampus, EC, LC and SN. In these areas, bacterial DNA profiles were dominated by a group of oral bacteria, mostly comprising those typical of subgingival populations, as seen in periodontitis (Socransky et al., 1998; Shi et al., 2018). The EC-C and LC-AD had less subgingival content (including an almost complete absence of *Porphyromonas*) and mainly taxa from origins defined as ‘uncharacterized’ by SourceTracker (Figure 9). LC-AD and EC-C population profiles were also distinct from each other and from all other groups in terms of OTU heatmap analysis (Figures 3A,B), alpha (Figure 5) and beta-diversity (Supplementary Figures 3AB, 4). The top 12 genera observed (Figure 7; Metastat analysis), mainly tended to show difference between areas rather than between disease and control. Interestingly, significantly differentially abundant individual taxa were largely confined to those in H-AD and LC-AD samples: as calculated by T-tests (31 genera; Supplementary Table S7), non-parametric Metastat analysis (21 genera; Figure 7; Supplementary Table S7) and LEfSe analysis (17 genera; Figure 8; Supplementary Table S7). Additionally, significant differences were seen between SN-C vs. SN-PD, EC-C vs. H-C and H-AD vs. H-C samples. Similarly, inter-group variance also demonstrated a significant difference between H-AD and LC-AD by every measure, however the other difference that was universally detected was between EC-C and SN-C (Supplementary Table S6).

#### Diversity: Origins of bacterial DNA found in the brain

The top 20 genera for each brain group and whole blood, as defined by 97% OTU clustering, are compared in Supplementary Table S10A,B with respect to oral content as defined by HOMD. We recently showed that the largest single contributor to the blood microbiome is the oral cavity (Emery et al., 2021); here, the top 20 bacterial genera in blood are 40.7% oral-derived. Of all brain groups, EC-C and LC-AD had the lowest oral content at 31.5 and 24.4% respectively, LC-PD had 59.6%, whilst SN-C (82.3%) and SN-PD (84.3%) had the highest oral content. SourceTracker analysis differed from this in two main respects: blood-associated bacterial content of brain groups was calculated at, on average, 63.5% which may be explained by the shared oral content of blood and brain samples, and lower predicted oral content, due to the use of a subgingival training data set rather than one from the whole oral cavity (as defined by HOMD). However, SourceTracker analysis is consistent with a pattern where EC-C and LC-AD differ from other areas. This includes higher levels of taxa from ‘uncharacterized’ sources in EC-C and LC-AD, explained by their *Cutibacterium* content which is likely to be primarily skin-derived, although also abundant in the nasal passages, with connections to the olfactory tract and LC.

### Comparison of brain sample bacterial DNA profiles with bacterial profiles in the oral environment

Studies of periodontal disease-specific changes in microbial composition have revealed associations between certain species (Socransky et al., 1998; Hajishengallis and Lamont, 2012). This has led to the classification of bacterial complexes, color-coded according to periodontal disease severity, describing a step-wise development from early colonizers of periodontal plaque, intermediate bridging complexes and finally late colonizers (red complex) strongly linked to the pathogenic state (Socransky et al., 1998). Subsequently, higher resolution data showed that these complexes contained larger numbers of different species with higher degrees of variability than previously catalogued. It was concluded that, rather than the presence or absence of pathogenic species, there is dysbiosis, whereby species already present, such as the red group taxa *P. gingivalis*, *Treponema denticola*, and *Tannerella forsythia*, adopt disease-related proportions relative to commensal species and altered phenotypes specific to the periodontal biofilm (Hajishengallis and Lamont, 2012).

More recently, different bacterial ecotypes have been associated with certain clinical stages of periodontal disease (Boutin et al., 2017), with ecotype-1 being more commonly associated with periodontal health and ecotype-2 mostly with disease in the oral cavity. Here, in oral-rich brain areas the three top OTUs with respect to LEfSe in ecotype-1, *Haemophilus parainfluenza* (*H. parainfluenza*), *Streptococcus* sp. and *Neisseria* sp. represented around 25% of sequence reads. Many of the other ecotype-1 taxa defined by LEfSe such as *Rothia* and *Corynebacterium*, were found at lower levels and were less consistent components. The top three ecotype-2 taxa, *Fusobacterium*, *Campylobacteria* and *Porphyromonas* represented around 11–12% of total OTUs, other components of ecotype-2, such as Bacteroidales, *Prevotella* and *Filifactor*, also featured, but *Treponema* was only found in four samples with only one above 1%. Interestingly, the mix of taxa (from both ecotypes-1 and -2) seen here in brain samples also resembles the spatially organized consortium of taxa within dental plaque (Mark Welch et al., 2016) which includes *Corynebacterium*, *Streptococcus*, *Porphyromonas*, *Haemophilus*/*Aggregatibacter*, Neisseriaceae, *Fusobacterium*, *Leptotrichia*, *Capnocytophaga*, and *Actinomyces*. Within this structure it is proposed that *Corynebacterium* acts as a nucleating component with an anchoring and framework role. *Corynebacterium* was a consistent component of the top 20 taxa seen here in brain samples, and clustered heatmap analysis shows it to be one of the taxa whose increased abundance is characteristic of LC-AD (Figure 3B). However, it is worth considering that even those bacteria associated with health in the mouth may not be benign when present within brain tissue. Likewise, the level of different types of bacteria present, may not be related to extent of detrimental outcome; even low levels of pathogenic bacteria, in the presence of ‘supporting’ bacteria may still provoke inflammatory outcomes. Finally, relevant to this present study, although not widely investigated, *C. acnes* (*aka P. acnes*) has been noted as being present in the oral cavity, is abundant in nasal passages (Achermann et al., 2014), and is among the microflora of endodontic infections, responsible for brain abscesses, endocarditis and other infections (Niazi et al., 2010).

#### Oral/periodontal bacterial species and AD

Historical evidence for an association between poor oral health and AD is now well established. Clinical studies have shown, for instance, that periodontitis is associated with increased incident MCI (Iwasaki et al., 2019), and a six-fold increased rate in cognitive decline (Ide et al., 2016). Furthermore, many studies support a significant association between periodontal disease and AD (review: Cerajewska et al., 2021), as highlighted in particular by a recent systematic review and large-scale cohort studies (Chen et al., 2017a; Choi et al., 2019; Hu et al., 2021). Additionally, increased levels of circulating antibodies to periodontal pathogens have been linked to an increased risk of AD and MCI (Noble et al., 2009; Sparks Stein et al., 2012; Noble et al., 2014). Large-scale population studies have also shown strong links between chronic periodontitis and dementia (Chen et al., 2017a). Studies have also linked specific measures of periodontal disease, such as periodontal pocket depth, alveolar bone loss, gum attachment loss and gingival bleeding with cognitive decline (Holmer et al., 2018; Nilsson et al., 2018). Periodontal disease has also been associated with higher Aβ load in the brains of the non-AD elderly and a reduced clearance of Aβ42 from cerebrospinal fluid (Kamer et al., 2021).

#### Oral/periodontal bacterial species and PD

A number of studies have also linked periodontitis to PD; this includes a large population-based cross-sectional investigation, with follow-up showing that professional mechanical plaque removal (PMPR) and root surface instrumentation (RSI) reduces the risk of PD (Chen et al., 2017b, 2018). Systemic inflammation is a major factor in PD progression (Kalia and Lang, 2015; Qin et al., 2016) with periodontitis as a significant putative contributor (Hajishengallis, 2015), with a possible mechanistic connection. Additionally, levels of *P. gingivalis*-derived gingipains, detected by antibody, were higher in PD blood compared to controls (Adams et al., 2019). In the present study, the highest levels of oral taxa of any brain area were in the SN with 82.3% in controls and 84.3% in PD samples.

### Locus coeruleus and its importance in AD and PD

This study demonstrated that the bacterial profile of the LC is distinct from other areas of the brain. The symptoms and pathology of both AD and PD have been associated with specific pathological changes in the brain’s isodendritic core (Rossor, 1981). This is the brain’s major noradrenergic source, projecting from the LC in the pons. It innervates, particularly the hippocampus and the cerebral cortex, but also the serotonergic dorsal raphe nucleus and dopaminergic SN in the midbrain, together with the cholinergic nucleus basalis of Meynert (nbM) in the basal forebrain, the amygdala, olfactory bulb, cingulate gyrus and, laterally, the cerebellum (Giorgi et al., 2019; Bari et al., 2020). This caudo-rostral direction of innervation generally parallels the direction of disease progression described in both AD and PD (Grinberg et al., 2011; Del Tredici and Braak, 2016), with the noradrenergic system, likely acting as a conduit for the spread of neurodegenerative pathology (Braak et al., 2011; Iba et al., 2015).

Variation can be seen between individuals. Some studies suggest that build-up of abnormal ptau often begins in the LC (Braak and Braak, 1991, 1995), and in other studies, the nbM (Mesulam et al., 2004), before reaching the trans entorhinal/entorhinal area (Braak et al., 2011; Elobeid et al., 2012). Others indicate that ptau pathology spreads to the LC from the transentorhinal area and EC (Kaufman et al., 2018), and it may be that the initial site of seeding of ptau is based on more than one initiating agent or event. Notably, the variations in the extent of disease in different brain regions may be related to the intrinsic health/genetic/immune status of individuals. Whatever the initiating event, pathology has been found in the LC at an early stage in both AD (Bondareff et al., 1981; Tomlinson et al., 1981; Forno, 1996; Braak et al., 2011; Theofilas et al., 2017) and PD (LBs) (Forno, 1996; Huynh et al., 2021). This disease pathology is associated with, not only neuronal impairment, axonal loss and subsequent reduction of noradrenaline delivery to target areas (Andres-Benito et al., 2017), but also increases vulnerability to neuropathology-associated damage in other areas.

Noradrenergic signaling is associated with reduction of inflammation; thus, its loss results in overproduction of inflammatory cytokines with resultant increase in amyloid production and its reduced clearance in both AD and PD (Feinstein et al., 2016; Giorgi et al., 2019, 2020). Loss of noradrenergic function also leads directly to loss of synaptic plasticity and reduced neuronal health, for instance, due to loss of NGF/BDNF, which exacerbates AD neural toxicity (Follesa and Mocchetti, 1993; Marien et al., 2004) in areas such as the hippocampus.

Pathology in the LC, although occurring early, until now has been seen to largely involve the presence of ptau, with very few amyloid plaques in this area until much later in the disease process. However, a recent study of the LC in AD patients (Kelly et al., 2021) shows an early appearance of amyloid largely as intraneuronal AβO or amyloid β-oligomers, a particularly active form of Aβ. This suggests that the AβO are likely to be responsible in a large part for activation of microglia in the LC, and may ultimately have a strong influence on the outcome of the pathology.

Neuronal damage to the LC is important in both early AD and PD pathology (Feinstein et al., 2002; Theofilas et al., 2017; Giorgi et al., 2020), and overlapping patterns of LC axonal damage might explain the differential presentation of symptoms in PD and AD (Del Tredici and Braak, 2013; Donaghy and McKeith, 2014; Vermeiren and De Deyn, 2017; Smith et al., 2019; Ferreira et al., 2020). AD and PD are known to share many symptoms associated with LC damage including stress, anxiety and disturbed sleep (German et al., 1992; Kelberman et al., 2020). Furthermore, loss of motor function in PD, associated with the basal ganglia also aligns with the loss of noradrenergic functional connectivity (Zhang et al., 2016).

In this study, statistical comparisons showed relatively few significant differences in bacterial populations between controls and disease within specified brain regions. However, LC-C and LC-PD groups did show substantial differences between health and disease: LC-C contained, on average 53.9% oral taxa and LC-AD had the lowest oral content at 24.4% on average. By contrast, LC-AD and LC-PD had substantially increased Actinobacteria, Cutibacteria which includes *C. acnes*-like sequences, which differentiated them from healthy controls.

### Oronasal taxa

The use of Novogene and Eurofins as two separate means of assessment, enabled consensus between bacterial species assignment; in the main, designations were in good agreement. As mentioned above, the strong presence of *Cutibacterium*, presented by Novogene as one of the top 4 species in every group of brain tissue examined, (ranging from 6.6–27.4%, Novogene; Supplementary Table S5), warranted further examination. *Cutibacterium* was the most prevalent bacterial genus in LC-AD, LC-PD and EC-C (but not LC-C). Notably, Eurofins analysis showed 164 different uncharacterized OTUs, of which their OTU1, comprised 87%. BlastN analysis attributed their OTU1 to *Cutibacterium*, *C. acnes* species.

Another group of OTUs are defined to (f) Propionibacteriaceae, but resemble *C. granulosum* and are substantially increased in the LC-PD sample. Of note, a proportion of *Cutibacterium* sequences found in the brain are discernibly different from those found in the NTC and may also be found in neurodegenerative disease-specific patterns. Interestingly, *C. acnes* has well characterized phylotypes with differing biological characteristics such as preferred niche, inflammatory properties and virulence factors (Mayslich et al., 2021). The distribution of bacteria, especially oral taxa and *Cutibacterium*, seen here may also shed light on possible entry points into the brain. Significantly different bacterial populations seen here in the EC (with lower oral and high *Cutibacterium* content) is consistent with the pathway of ptau deposition (Braak and Braak, 1995), starting in the EC and transentorhinal cortex; this led to a proposal that microbial incursion could occur *via* the nasal cavity; further, evidence for the naso-pharyngeal route of entry of various bacteria into the brain has been described (Leheste et al., 2017). Notably, the sinonasal microbiome shows considerable overlap with the EC populations seen here and contains major *C. acnes* and *C. granulosum* components (Earl et al., 2018).;

The association of *Cutibacterium* with opportunistic infection is well documented (Leheste et al., 2017) and has been found not only in degenerative spinal tissue and other peripheral sites but, also in the brain (Zaffiri et al., 2013; Burnham et al., 2014; Emery et al., 2017; Capoor et al., 2021) including AD brain (Kornhuber, 1996; Emery et al., 2017). A severe skin disorder, *acne inversa* has also been associated with AD (Wang et al., 2010). *C. acnes* in intraoperative spinal tissue has also been associated with early-onset PD (Mourad et al., 2021) and *C. acnes* clusters have been found in midbrain axons of PD donors (Leheste et al., 2017).

## Conclusion

In this study, samples of tissue from different areas of the brain were obtained post-mortem from AD, PD and control donors, and assessed for microbial content using PCR-based 16SNGS. The majority of the bacteria detected are associated with oronasal origin. Firstly, although our recent NGS examination of the blood microbiome, showed that the bacterial content was overwhelmingly oral in nature, notably, the bacterial profile of blood does not substantially overlap with the brain regions examined, suggesting that the brain microbiome is not likely to originate predominantly due to simple passage through the BBB.

Secondly, many studies support a significant association between periodontal disease and AD, and also for PD, although the data is less well developed. As can be seen in this present study, the most prevalent bacterial species are from consistent combinations of oral (and probably oronasal) taxa. With the qualification that these are small donor numbers and that pre-mortem infections can drastically alter the bacterial composition, we see here, for instance, that all the brain areas, with the particular exception of EC-C and LC-AD, noticeably contain the oral bacteria *Streptococcus*, *Haemophilus*, *Prevotella*, *Prevotella 7*, *Fusobacterium*, *Porphyromonas*, *Neisseria*, *Campylobacter*, and *Alloprevotella* as mainly present within their top 5–10 bacterial prevalence. The data also shows that the SN contains the highest levels of oral taxa of all areas examined.

Thirdly, the genus *Cutibacterium*, although not seen here in blood, consistently has a place within the first four highest prevalent bacteria in all brain areas examined, notably being in the highest position in EC-C, LC-C, and LC-PD. The data shown here suggests that, in addition to subgingival pathobionts, some disease-specific distribution was also seen with *C. acnes*-related and *C. granulosum*-related sequences. Along with the subgingival bacterial group, these differences were most extreme in the LC. Notably, as mentioned above, there is a direct projection of the olfactory bulb to the EC, and also an LC projection to the olfactory bulb in which the nasal passage/olfactory tract is dominated by bacteria such as *Cutibacterium*, e.g., *C. acnes* (Achermann et al., 2014). Since neuropathological studies show that the LC, EC and trans/EC are putative points of pathology initiation for AD and PD, given the data presented here, we suggest that bacterial infiltration, perhaps involving a combination of *Cutibacterium* and oral species such as *Streptococcus* or *Staphylococcus*, may result in the instigation or exacerbation of neuroinflammation and subsequent neuropathology.

Fourthly, the assessment, in this present study, of the range of microbial presence in different brain areas in AD and PD compared with control, needs to be considered in the knowledge of the known roles of Aβ and α-synuclein as antimicrobial peptides (AMPs) and their ability to escalate neuroinflammation in the presence of microbes. The difference in microbial content of the EC and LC, compared with the other brain areas, may relate to their additional access to the olfactory tract. Even if the microbial content, noted here in the LC and other brain areas, are not initiating factors but exacerbators, their presence and their putative effects over a number of years/decades need to be considered regarding the neurodegenerative effects in AD and PD.

Finally, the microbiological profiles examined here are from one main cohort based in the Bristol area, all samples were taken from the SWDBB. It is undeniable that the microbiological profiles from any one NGS-based study or methodology will always be incomplete with respect to the global human microbiome, and an additional qualification to this is that these are small donor numbers and that pre-mortem infections can drastically alter the bacterial composition. Nevertheless, from this study, it is observable that many bacteria populate AD, PD and apparently normal brains in all areas examined. Certain bacteria predominate, in general these seem to be oral or oronasal-derived. Yet to be determined is which of these have adverse and which have no effects; why their spread is apparently so slow and what is their importance in relation to other possible co-habitants such as viruses and fungi as reported by others.

## Data availability statement

This Targeted Locus Study project has been deposited at DDBJ/EMBL/GenBank under the accession KFLD00000000. The version described in this paper is the first version, KFLD01000000.

## Ethics statement

The studies involving human tissue were reviewed and approved by the South West Dementia Brain Bank (SWDBB) and National Research Ethics Service (NRES). South West Cornwall, Plymouth Research Ethics Committee (approval 13/SW/0272 renewal 18/SW/0029). Individuals provided their written informed consent to donate their tissue to the Brain Bank for research into neurodegenerative disease.

## Author contributions

DE, TC, JT, MH, AP, MD, SA-B, and NW all helped in the conception of the study and design of the experimental approach. DE organized collection of data. DE, MD, TC, JT, and MH collected the data, performed laboratory work, and analysed the output data. AP contributed to data handling and analysis. Ongoing analysis of data was discussed by all participants. DE, SA-B, NW, MD, and TC all contributed to drafting the manuscript. All authors contributed to the article and approved the submitted version.

## Funding

We are most grateful to GlaxoSmithKline, for their equitable donation to support our work on Alzheimer’s disease and bacteraemia. The funder was not involved in the study design, collection, analysis, interpretation of data, the writing of this article or the decision to submit it for publication. This was the sole source of funding. Tissue for this study was provided with support from the BDR programme, jointly funded by Alzheimer’s Society, United Kingdom and Alzheimer’s Society. The SWDBB is further supported by Bristol Research into Alzheimer’s and Care of the Elderly (BRACE)’. The University of Bristol acted as Research Sponsor from 04.12.2013, until 30.7.2019 with an HRA approved extension to 31.12.2020.

## Conflict of interest

The authors declare that the research was conducted in the absence of any commercial or financial relationships that could be construed as a potential conflict of interest.

## Publisher’s note

All claims expressed in this article are solely those of the authors and do not necessarily represent those of their affiliated organizations, or those of the publisher, the editors and the reviewers. Any product that may be evaluated in this article, or claim that may be made by its manufacturer, is not guaranteed or endorsed by the publisher.

## Footnotes

^1^ http://ccb.jhu.edu/software/FLASH/

^2^ http://qiime.org/

^3^ http://drive5.com/uparse/

^4^ http://www.arb-silva.de/

^5^ http://www.drive5.com/muscle/

^6^ homd.org

^7^ https://gmrepo.humangut.info/data/project/PRJEB6092

^8^ https://www.ncbi.nlm.nih.gov/bioproject/PRJNA419453

^9^ http://qiime.org/

^10^ http://www.mothur.org/wiki/Amova

^11^ https://cran.r-project.org/web/packages/vegan/vegan.pdf

## Supplementary material

SUPPLEMENTARY FIGURE 1 Samples and sequence reads **(A)** Species accumulation curve displayed as a boxplot **(B)** Summarised total sequence processing, read merging, Operational Taxonomic Units (OTU), sequence and MED OTU statistics

SUPPLEMENTARY FIGURE 2 Clustal Omega Alignment Analysis of uncharacterised *Cutibacterium* OTUs. This indicates notable clustering of AD exclusive OTUs. AD, Alzheimer’s disease; PD, Parkinson’s disease. ALL-AD; PD; Control and NTC; EC, entorhinal cortex; NTC, no template control.

SUPPLEMENTARY FIGURE 3 Beta diversity boxplots depicted by **(A)** unweighted Unifrac and **(B)** Unifrac weighted by phylogenetic distance, both showing significant differences between groups especially pairings including EC-C or LC-AD.

SUPPLEMENTARY FIGURE 4 Beta diversity Heatmap (weighted Unifrac distance). Heat map showing the beta diversity dissimilarity coefficients between brain samples.

SUPPLEMENTARY FIGURE 5 **(A)** Real-time PCR displayed to compare between controls and disease and **(B)** comparison between samples of ≤ 40 and > 40 h post mortem interval (PMI). Brain regions did not show significant effect on 16S rRNA DNA levels with disease state or post-mortem interval (PMI) except for the temporal BA21/22 area, which had increased bacterial DNA levels in those with PMIs > 40 h PMI. AD, Alzheimer’s disease; C, Control (no dementia); PD, Parkinson’s disease; BA, Brodmann area; PMI, post mortem interval. Human brain areas: anterior temporal cortex BA38 (AT), entorhinal cortex BA34 (EC), hippocampus (H), locus coeruleus (LC), orbito-frontal/ (lower)pre-frontal cortex BA11 (LF), substantia nigra pars compacta (SN), dorsolateral cortex (BA9), brain stem (BS, area below LC), middle temporal cortical gyrus (BA21/22) and nucleus basalis of Meynert (nbM).
