# Supplementary figures and images for: High resolution 16S rRNA gene Next Generation Sequencing study of brain areas associated with Alzheimer’s and Parkinson’s disease
Source: Front Aging Neurosci. 2022 Dec 9;14:1026260. doi: 10.3389/fnagi.2022.1026260 (PMC9780557; doi:10.3389/fnagi.2022.1026260)

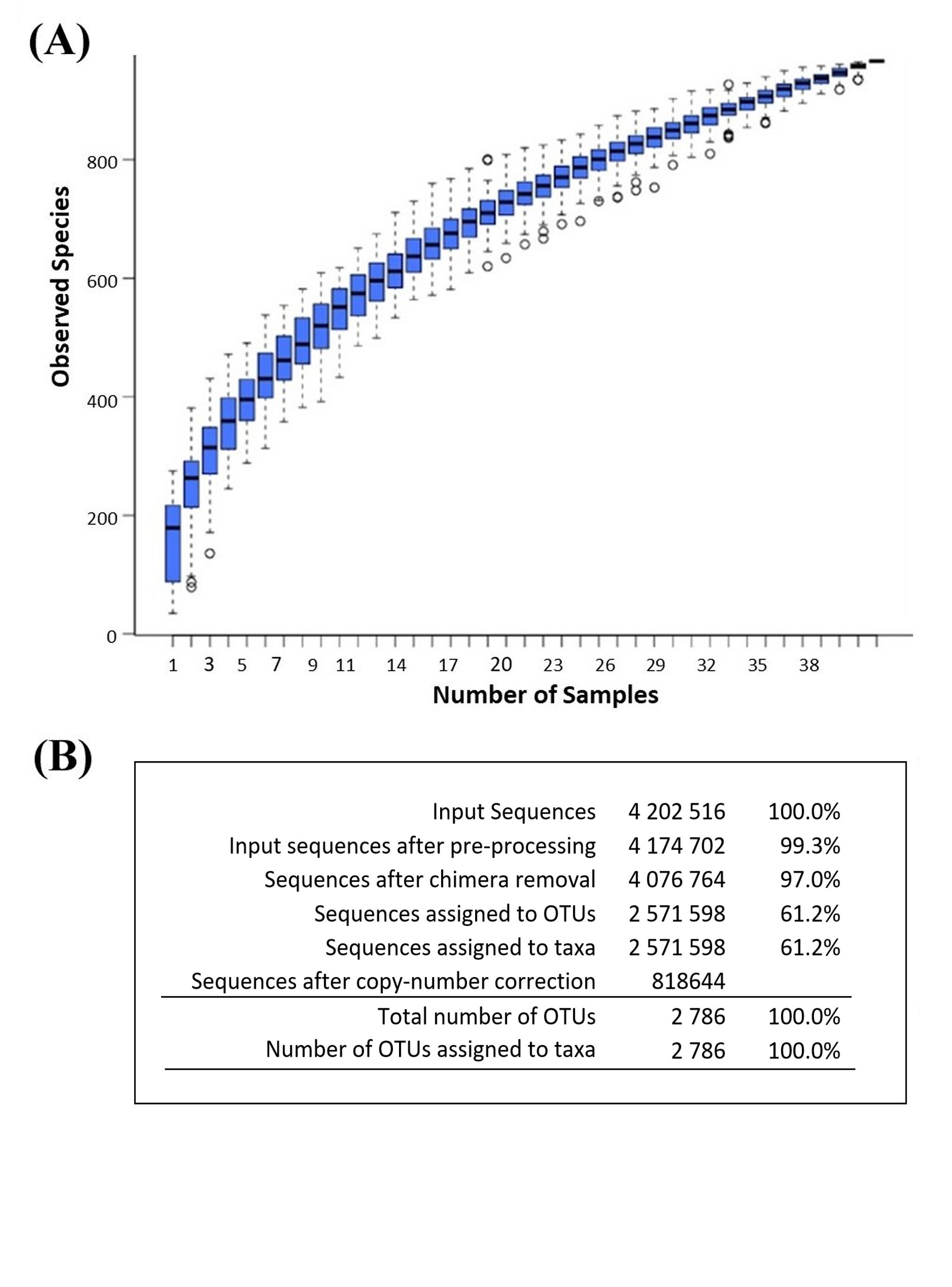

Supplement: SUPPLEMENTARY FIGURE 1 — Samples and sequence reads (A) Species accumulation curve displayed as a boxplot (B) Summarised total sequence processing, read merging, Operational Taxonomic Units (OTU), sequence and MED OTU statistics [file Image_1.jpeg]

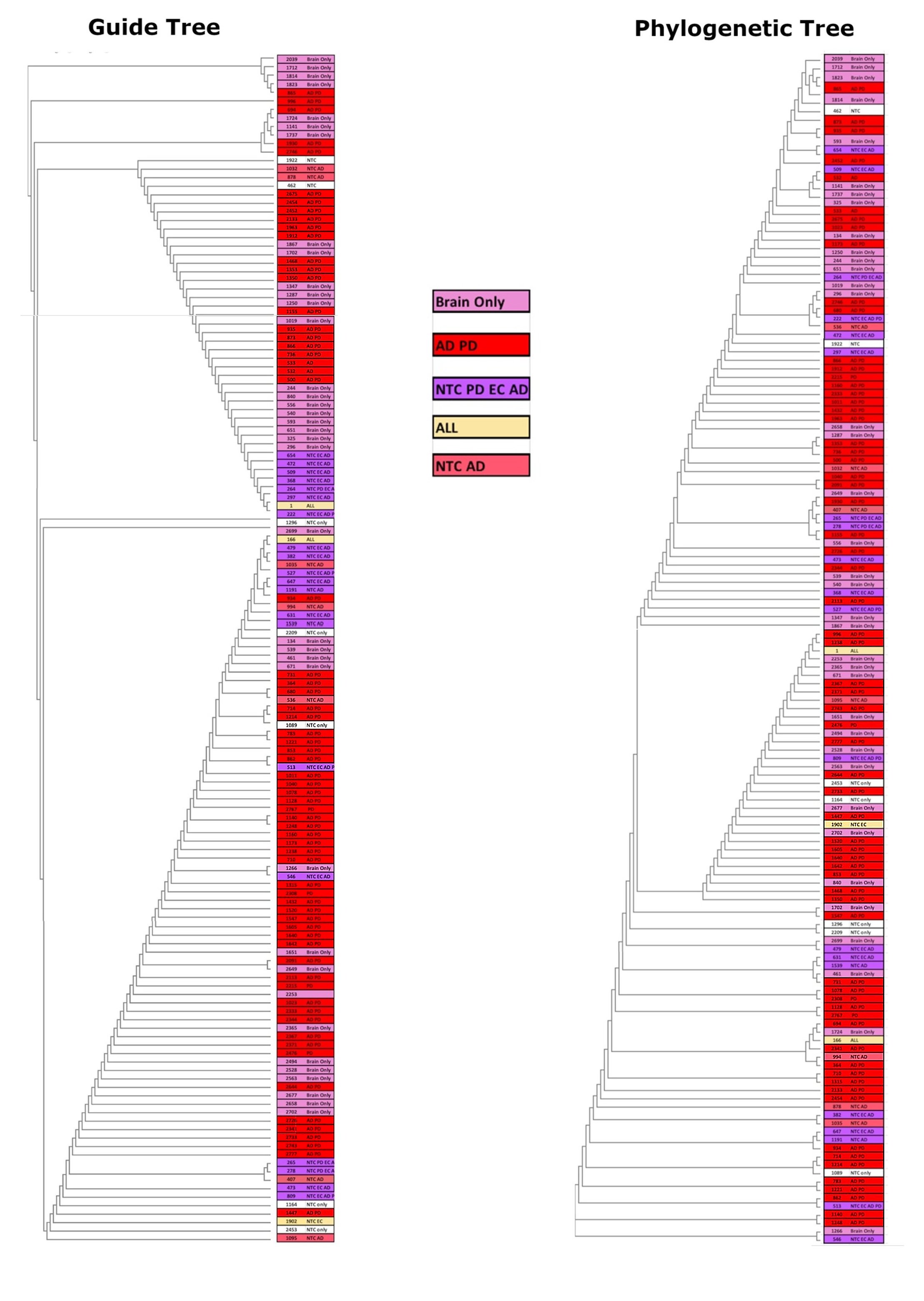

Supplement: SUPPLEMENTARY FIGURE 2 — Clustal Omega Alignment Analysis of uncharacterised Cutibacterium OTUs. This indicates notable clustering of AD exclusive OTUs. AD, Alzheimer’s disease; PD, Parkinson’s disease. ALL-AD; PD; Control and NTC; EC, entorhinal cortex; NTC, no template control. [file Image_2.jpeg]

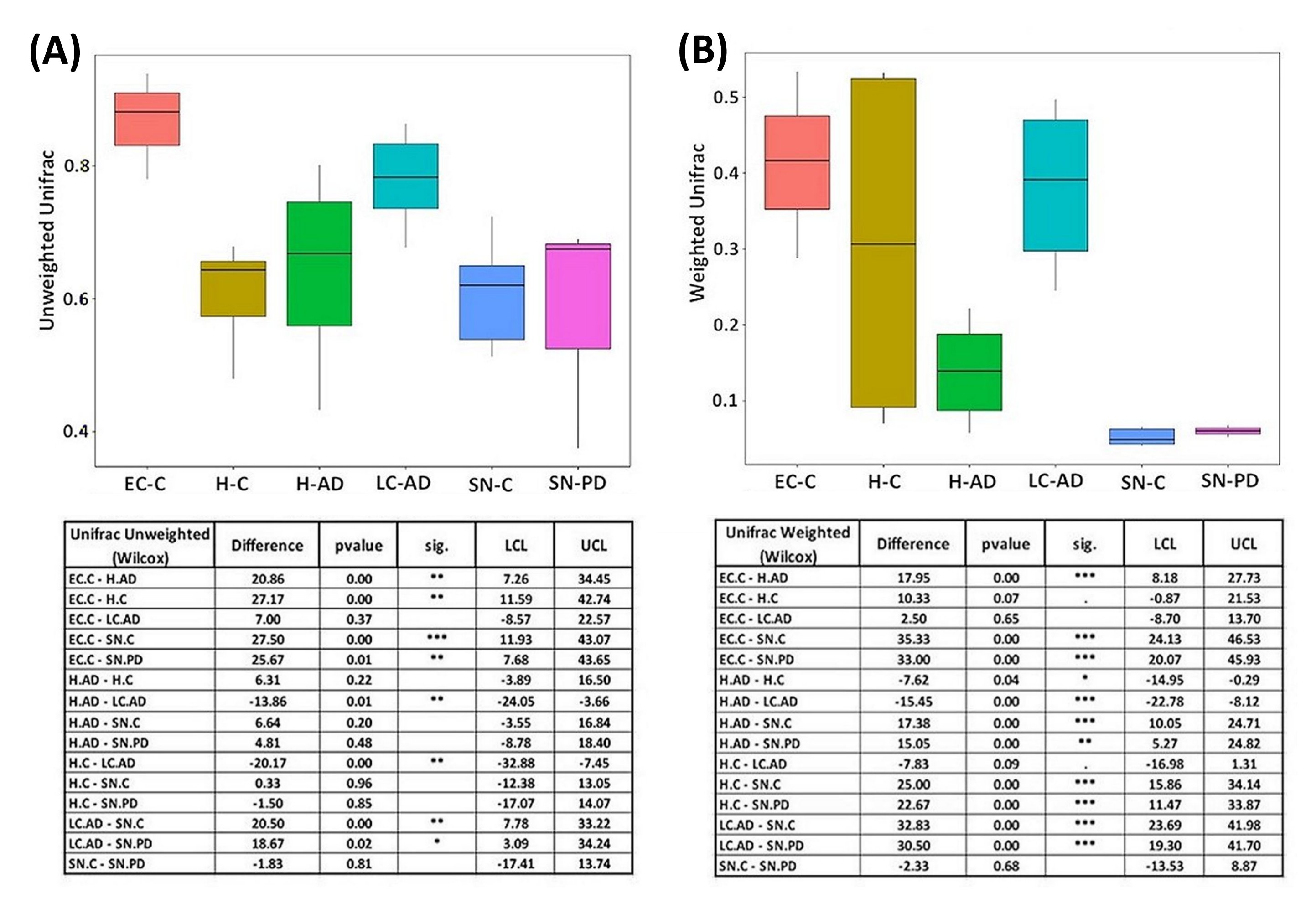

Supplement: SUPPLEMENTARY FIGURE 3 — Beta diversity boxplots depicted by (A) unweighted Unifrac and (B) Unifrac weighted by phylogenetic distance, both showing significant differences between groups especially pairings including EC-C or LC-AD. [file Image_3.jpeg]

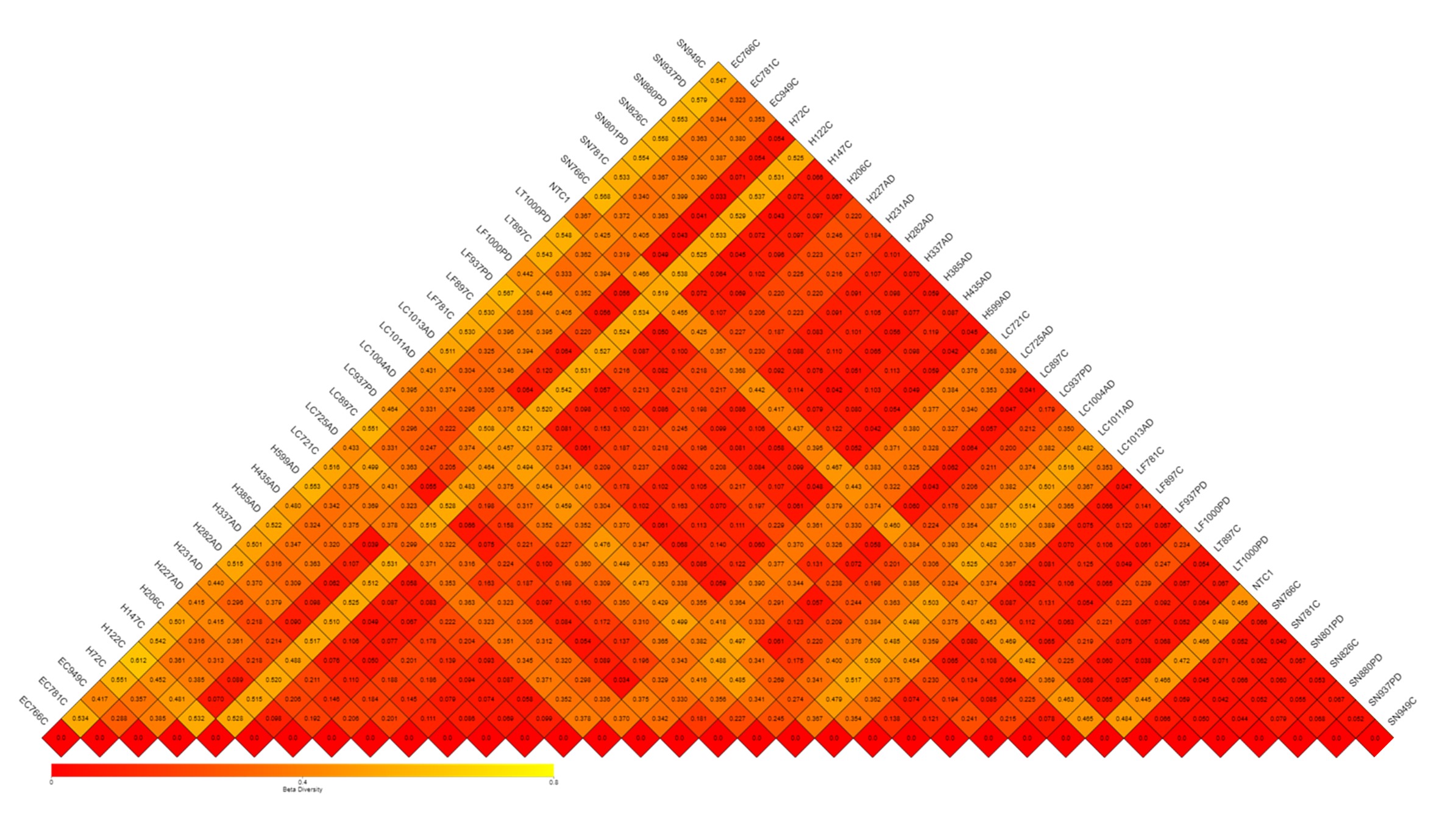

Supplement: SUPPLEMENTARY FIGURE 4 — Beta diversity Heatmap (weighted Unifrac distance). Heat map showing the beta diversity dissimilarity coefficients between brain samples. [file Image_4.jpeg]

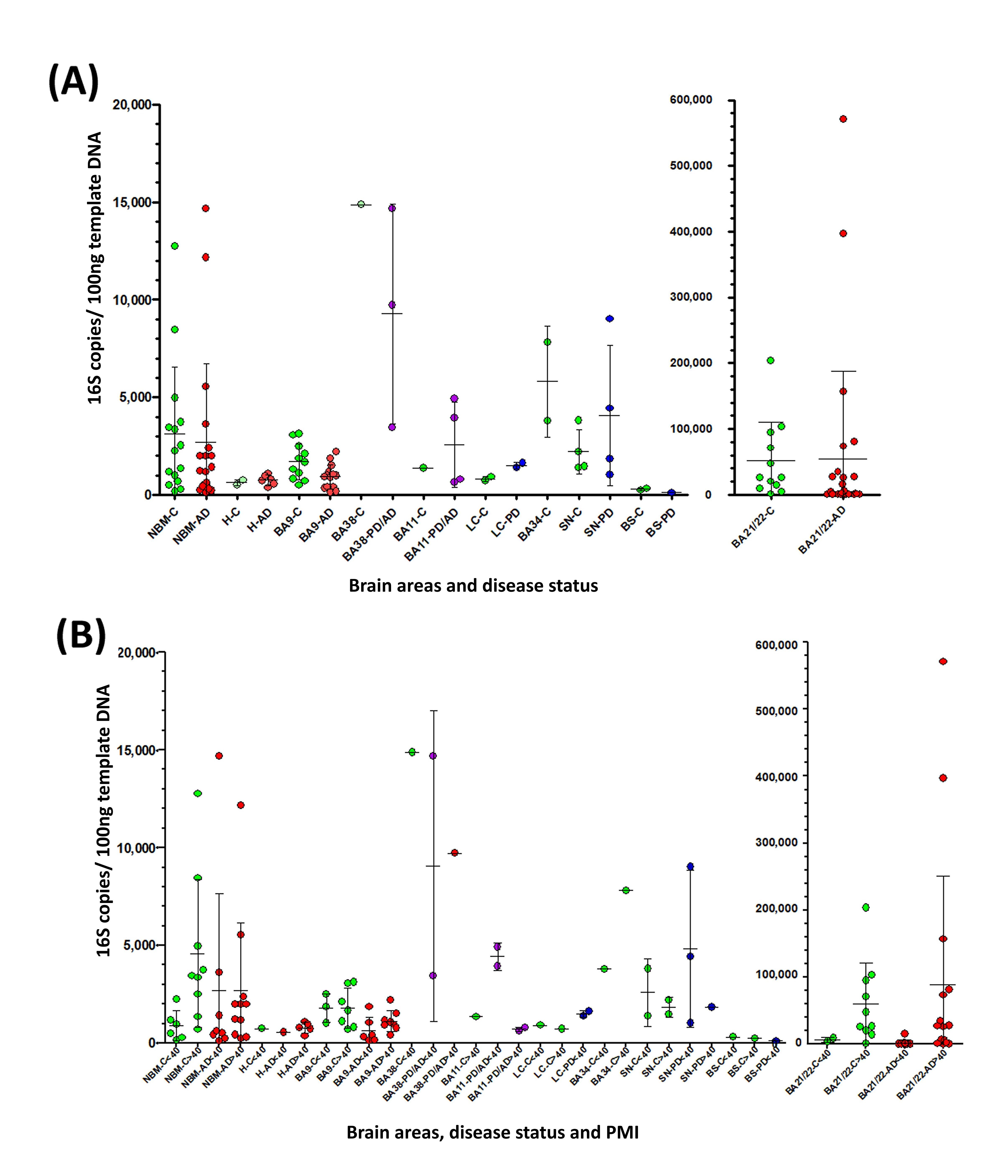

Supplement: SUPPLEMENTARY FIGURE 5 — (A) Real-time PCR displayed to compare between controls and disease and (B) comparison between samples of ≤ 40 and > 40 h post mortem interval (PMI). Brain regions did not show significant effect on 16S rRNA DNA levels with disease state or post-mortem interval (PMI) except for the temporal BA21/22 area, which had increased bacterial DNA levels in those with PMIs > 40 h PMI. AD, Alzheimer’s disease; C, Control (no dementia); PD, Parkinson’s disease; BA, Brodmann area; PMI, post mortem interval. Human brain areas: anterior temporal cortex BA38 (AT), entorhinal cortex BA34 (EC), hippocampus (H), locus coeruleus (LC), orbito-frontal/ (lower)pre-frontal cortex BA11 (LF), substantia nigra pars compacta (SN), dorsolateral cortex (BA9), brain stem (BS, area below LC), middle temporal cortical gyrus (BA21/22) and nucleus basalis of Meynert (nbM). [file Image_5.jpeg]
